# Metatranscriptomic analysis to define the Secrebiome, and 16S rRNA profiling of the gut microbiome in obesity and metabolic syndrome of Mexican children

**DOI:** 10.1186/s12934-020-01319-y

**Published:** 2020-03-06

**Authors:** Luigui Gallardo-Becerra, Fernanda Cornejo-Granados, Rodrigo García-López, Alejandra Valdez-Lara, Shirley Bikel, Samuel Canizales-Quinteros, Blanca E. López-Contreras, Alfredo Mendoza-Vargas, Henrik Nielsen, Adrián Ochoa-Leyva

**Affiliations:** 1 Departamento de Microbiología Molecular, Instituto de Biotecnología, Universidad Nacional Autónoma de México, Avenida Universidad 2001, C.P. 62210 Cuernavaca, Morelos Mexico; 2 Unidad de Genómica de Poblaciones Aplicada a la Salud, Facultad de Química, UNAM/Instituto Nacional de Medicina Genómica (INMEGEN), México City, Mexico; 3 Instituto Nacional de Medicina Genómica, Secretaría de Salud, México City, Mexico; 4 Section for Bioinformatics, Department of Health Technology, Technical University of Denmark, Kgs. Lyngby, Denmark

**Keywords:** Secrebiome, Microbiota, Microbiome, Obesity, Metabolic syndrome, Metatranscriptome, Metatranscriptomics, AAR, CAZY, Secretome

## Abstract

**Background:**

In the last decade, increasing evidence has shown that changes in human gut microbiota are associated with diseases, such as obesity. The excreted/secreted proteins (secretome) of the gut microbiota affect the microbial composition, altering its colonization and persistence. Furthermore, it influences microbiota-host interactions by triggering inflammatory reactions and modulating the host's immune response. The metatranscriptome is essential to elucidate which genes are expressed under diseases. In this regard, little is known about the expressed secretome in the microbiome. Here, we use a metatranscriptomic approach to delineate the secretome of the gut microbiome of Mexican children with normal weight (NW) obesity (O) and obesity with metabolic syndrome (OMS). Additionally, we performed the 16S rRNA profiling of the gut microbiota.

**Results:**

Out of the 115,712 metatranscriptome genes that codified for proteins, 30,024 (26%) were predicted to be secreted, constituting the Secrebiome of the gut microbiome. The 16S profiling confirmed an increased abundance in Firmicutes and decreased in Bacteroidetes in the obesity groups, and a significantly higher richness and diversity than the normal weight group. We found novel biomarkers for obesity with metabolic syndrome such as increased Coriobacteraceae, *Collinsela*, and *Collinsella aerofaciens*; Erysipelotrichaceae, *Catenibacterium* and *Catenibacterium sp.*, and decreased *Parabacteroides distasonis*, which correlated with clinical and anthropometric parameters associated to obesity and metabolic syndrome. Related to the Secrebiome, 16 genes, homologous to *F. prausniitzi*, were overexpressed for the obese and 15 genes homologous to Bacteroides, were overexpressed in the obesity with metabolic syndrome. Furthermore, a significant enrichment of CAZy enzymes was found in the Secrebiome. Additionally, significant differences in the antigenic density of the Secrebiome were found between normal weight and obesity groups.

**Conclusions:**

These findings show, for the first time, the role of the Secrebiome in the functional human-microbiota interaction. Our results highlight the importance of metatranscriptomics to provide novel information about the gut microbiome’s functions that could help us understand the impact of the Secrebiome on the homeostasis of its human host. Furthermore, the metatranscriptome and 16S profiling confirmed the importance of treating obesity and obesity with metabolic syndrome as separate conditions to better understand the interplay between microbiome and disease.

## Background

The gut is an essential metabolic, endocrine, and immune organ inhabited by millions of microbes. Recently, our knowledge of the diversity of microorganisms in the human gut has exponentially increased with the development of high-throughput sequencing technologies [1]. The intestinal microorganism community (gut microbiota) is a dynamic ecosystem with critical functional roles in the development and physiology of its host, preventing the growth of pathogenic bacteria, modulating the immune response, affecting nutrient absorption, regulating metabolic processes, etc [1]. Meanwhile, the host’s intestinal microenvironment also impacts the structure and function of the microorganisms which inhabit it [2, 3]. Although metagenomic approaches provide information on the microbiota composition and the potential functions of the codified genes, the expression profile of the community is needed to know what genes are expressed under certain conditions [4, 5].

High-throughput sequencing of RNA transcripts (RNA-seq) from microbial communities (metatranscriptome) allows an unprecedented opportunity to analyze the functional and taxonomical dynamics of the expressed microbiome, which can be associated with human health and disease [1]. The microbial encoded genes are not directly correlated with their transcription; up to 41% of microbial transcripts in the human gut have different relative abundances as compared to their genome content [4]. Metatranscriptome analysis has been applied to the human gut microbiota, revealing changes on the microbial community gene-expression profile during the exposure to dietary [6] and xenobiotic interventions [7] or with the presence of inflammatory diseases [8], showing divergent expression profiles of microbial community (subject-specific expression) [9], as well as a core of expressed functions [10, 11]. Recently, a metatranscriptomic ‘core’ universally transcribed over time and across participants, often by different microorganisms, has been identified [3]. Thus, metatranscriptomics emerges as a highly informative approach to analyze the expressed functional dynamics of microbial communities that can be associated with the presence of diseases [1].

The secretome is defined as the complete set of Excreted/Secreted (ES) proteins of a cell [12]. These proteins play a critical role in biological processes important for gut colonization, persistence, interaction with mucosal cells, activating signaling pathways, contributing to probiotic effects, etc. In pathogenic bacteria, the secretome plays a crucial role in parasitism, modulating the host immune response and promoting the proliferation of infection [13–15]. Millions of years of co-evolution between the human host and its gut microbial community have resulted in a continuous dialogue of the microbiota with the immune system promoting the gut homeostasis. Thus, the collection of secreted proteins represents proteins released by the microbiota into the intestinal lumen for bacteria-bacteria and bacteria-host interactions regulating the interaction within the gut. However, current knowledge of the molecular mechanisms responsible for the cross-talk between the gut microbiota and the human host remains incomplete.

There are several examples of the role that ES proteins from the microbiota play in the gut’s health. Bile salt hydrolases of lactobacilli reduce blood cholesterol and diminish the risk for cardiovascular diseases [16, 17], and show activity against intestinal protozoan parasites [18]. Other enzymes such as N-acylated homoserine lactone (AHL)-lactonase help modulate the structure of the microbiota by decreasing the quorum-sensing of pathogenic bacteria [19]. Several secreted proteins act as essential mediators for the establishment of a bifidobacteria-host immune system dialogue [20]. However, no study has been conducted to examine the total secretome of the gut microbiota, and most importantly, what is the expressed secretome codified in the metatranscriptome of the human gut microbiome. Thus, the primary goal of this study was to determine the expressed secretome of the human gut microbiome associated with childhood obesity.

An essential step for the energy harvesting by the microbiota involves the carbohydrate degradation from food. Microbial enzymes conduct the breakdown of the diet’s complex oligosaccharides into fermentable monosaccharides and their posterior transformation into components that can be absorbed by the intestinal cells and the microbiota [21]. Given that the human genome lacks the enzymes required to cleave the glycosidic bonds of the complex dietary polysaccharides [22], this process is mainly performed by three types of Carbohydrate active enzymes (CAZymes) enzymes: the Carbohydrate Esterases (CEs), Glycoside Hydrolases (GH), and Polysaccharide Lyases (PL). CAZymes plays an essential role in determining the nutritional status of individuals. The carbohydrate degradation resulting from the bacterial fermentation of fiber-rich diets constitutes the first step in the production of short-chain fatty acids (SCFAs), essential molecules generally associated with multiple health-promoting and beneficial metabolic effects for the host. However, the increased energy harvest derived by this process has also been proposed to contribute to diet-induced obesity in mice [23].

Here, we report a deeply sequenced metatranscriptome study of human gut microbiome samples to elucidate the functional profile of the Secrebiome. We carried out a high throughput shotgun sequencing of the total RNA and a profiling of the 16S rRNA gene using the V4 region as a marker for the phylogenetic diversity of the expressed bacterial community. Then, we constructed a de novo metatranscriptome to assess the total bacterial genes expressed and secreted that are present in the different groups. Using high-throughput sequencing, we determined for the first time, that ~ 26% of the total genes expressed in the metatranscriptome corresponded to potentially secreted proteins, which may serve as a critical mechanism to regulate the structure–function of the microbiome and their relationship with the host. Furthermore, 31 of these genes were differentially expressed in the obese (O) and obese with metabolic syndrome (OMS) groups. We also observed some novel correlations between differentially abundant taxa and the clinical and anthropometric parameters of the children cohort. When characterizing the Secrebiome, we observed an elevated enzymatic activity, mostly from hydrolases and transferases, which suggests the critical role that these proteins have in the metabolism of nutrients present in the host system.

## Results

### Different microbiota structures were found in normal weight and obesity groups

The study population of this work consisted of 27 children, classified as follows: 7 normal weight (NW), 10 with obesity (O), and 10 with obesity and metabolic syndrome (OMS) selected from a cohort of 750 children around 9 years of age. From the cohort, 333 samples were collected for RNA extraction, where 65% had normal weight, and the remaining 35% were obese. Importantly, 52.94% of obese children also had metabolic syndrome. Anthropometric and biochemical characteristics of the population are shown in Additional file 1: Table S1. Most of anthropometric and clinical parameters related to the obesity and obesity with metabolic syndrome were statistically different among the NW, O and OMS groups (Additional file 1: Table S1).

**Fig. 1:**
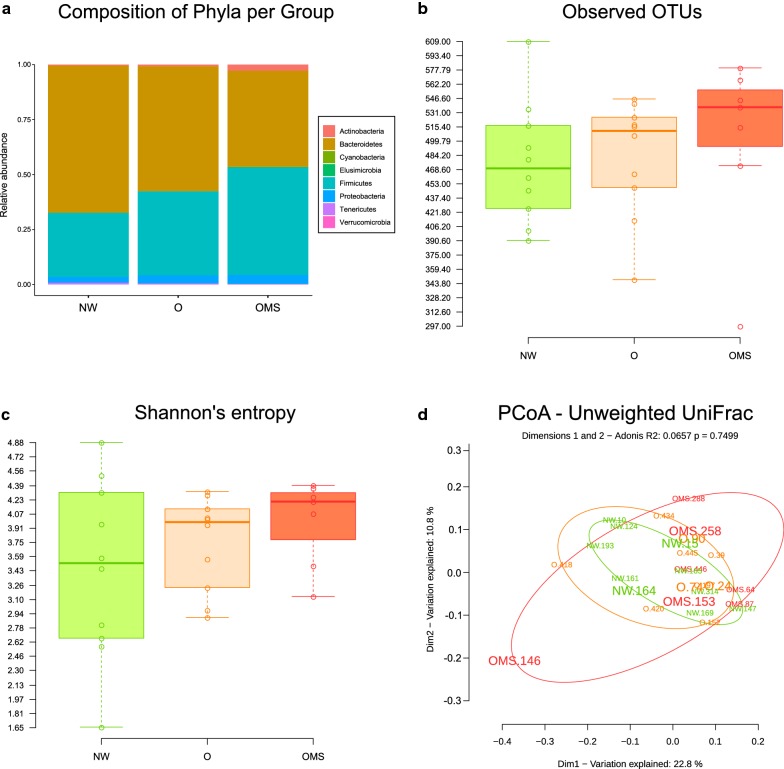
Analysis of the 16S rRNA gene profiles. Each panel compares the NW, O, and OMS groups. **a** Mean Relative abundance of the phyla present in each group. **b** Mean observed OTUs per sample and group. The averaged total unique OTUs from 10,000 rarefactions per sample are shown as points with overlying boxplots showing the distribution within each group. **c** Mean Shannon’s entropy per sample and group. The averaged Shannon’s index value from 10,000 rarefactions per sample are shown as points with overlying boxplots showing the distribution within each group. **d** Principal coordinate analyses of Unweighted UniFrac distances. Elipses were calculated based on the most distant samples per group. Samples for which RNA-seq information is available are presented with a larger font

For the 16S profiling of the microbiota associated with the three groups, we sequenced the corresponding V4 region amplicons of the 16S rRNA gene, and after the application of quality filters, we obtained 2,937,796 joined reads that were classified into 801 OTUs at 97% sequence identity. All samples successfully recovered most of the groups variation, as seen in the rarefaction curve, which shows saturation of diversity at a sequence depth of 20,000 reads (Additional file 2: Fig. S1a, b). Regarding the associated taxonomy of all samples, the bulk of OTU abundance showed that the three groups are dominated by the same three phyla Firmicutes, Bacteroidetes, and Proteobacteria, accounting for 39%, 56%, and 3% of the total reads, respectively (Fig. 1a and Additional file 3: Fig. S2a–f). Firmicutes was markedly increased in the OMS (46.44%), and O (40.29%) as compared to the NW group (26.65%), although only the difference between NW and OMS was statistically significant (p = 0.0431). The opposite effect was observed with Bacteroidetes, which was increased in NW (66.88%) as compared to the O (55.27%) and OMS (45.48%), although only the difference between the NW and OMS was significant (p = 0.0249). Interestingly, the ratio of Firmicutes/Bacteroidetes was NW = 0.55, O = 0.92, and OMS = 1.20, with significance difference only between NW Vs OMS (p = 0.025). Furthermore, both the OMS and O groups exhibited significantly higher richness and diversity than the NW group, with the larger values in the OMS group (Fig. 1b, c). The between-sample diversity comparison carried out with a PCoA ordination based on unweighted UniFrac distances showed no defined clusters by group (R^2^ = 0.066, p = 0.75), although the most disperse one was the OMS (based on the within-group variation), followed by the O and NW groups (Fig. 1d). The weighted distances showed the same behavior (Additional file 4: Fig. S3).

**Fig. 2:**
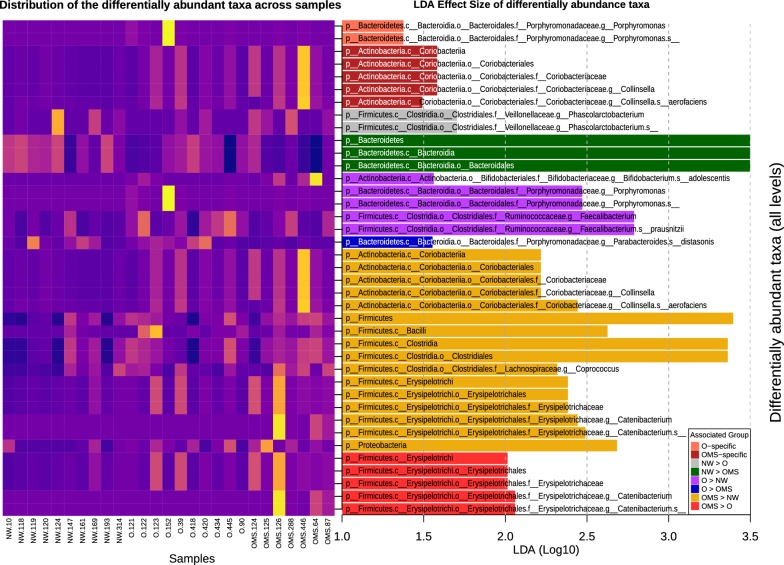
Differentially abundant taxa in association with groups and their distribution in the samples. Bars show the effect size of taxa that are differentially more abundant in the specified groups after analysis with LEfSe. Those labeled as specific were found significantly more in their groups, whereas the rest are found in pairwise group comparisons. The heatmap shows the abundance of those taxa in the samples with more abundant taxa in yellow

We found significant differences in the abundance of 41 taxa at different taxonomic levels between the three groups (Fig. 2). The class Coriobacteria, order Coriobacteriales, family Coriobacteraceae, genus *Collinsella* and species *Collinsella aerofaciens* were significantly more abundant specifically in the OMS group (when compared against both the NW and O groups), thus suggesting them as potential biomarkers for obesity with metabolic syndrome. Likewise, the genus *Porphyromonas* and, more specifically, an undetermined species in the same genus were significantly more abundant specifically in the O group when compared against NW and OMS, suggesting them as potential biomarkers for obesity.

Regarding pairwise group statistical significant comparisons, the genus *Phascolarctobacterium* and one undetermined species within this genus were more prevalent in the NW as compared to O, while Bacteroidetes, Bacteroidia, and Bacteroidales were more abundant in the NW than in the OMS group (Fig. 2). The O group showed an over-abundance of bacteria from the *Porphyromonas* and *Faecalibacterium* genera, as well as *Faecalibacterium prausnitzii*, *Bifidobacterium adolescentis*, and an undetermined species of *Porphyromonas* as compared to NW, while *Parabacteroides distasonis* was over-abundant in the O than in the OMS (Fig. 2). The OMS group showed a larger number of differentially abundant taxa at different levels, with the class Erysipelotrochi, order Erysipelotrichales, family Erysipelotrichaceae, genus *Catenibacterium* and an undetermined *Catenibacterium* species reported more over-abundant in the OMS group than in either the NW or O groups when considered separately (Fig. 2). Additionally, the OMS had over-abundance of phyla Firmicutes and Proteobacteria; class Coriobacteriia, Bacilli, and Clostridia; order Coriobacteriales and Clostridiales; family Coriobacteriaceae, genera *Colinssella*, and *Coprococcus*; and species *Colinsella aerofaciens* when compared to NW (Fig. 2).

### Microbiota changes are associated with anthropometric and clinical parameters

**Fig. 3:**
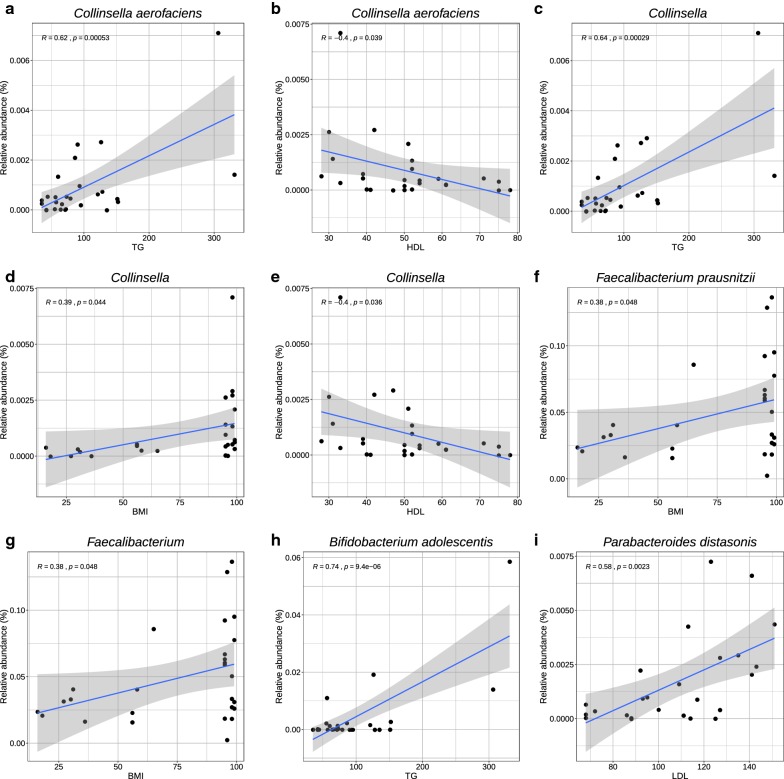
Linear regression and Pearson correlation of LEfSe biomarkers. The title of each graph corresponds to the LEfSe biomarkers taxa. The x-axis shows the value of the clinical and anthropometrical parameters, and the y-axis shows the relative abundance for each taxa

We analyzed whether the 41 differentially abundant taxa were correlated with changes in the anthropometric and clinical parameters that are involved in obesity and metabolic syndrome (Additional file 5: Table S2). Interestingly, *Collinsella aerofaciens* which was over-abundant in OMS vs all groups, showed a significant positive correlation with triglycerides (r = 0.62, p = 0.00053) (Fig. 3a), while showing a negative correlation with HDL (r = − 0.4, p = 0.039) (Fig. 3b). Interestingly, *C. aerofaciens* also showed a positive, albeit weak, correlation with waist circumference (r = 0.28, p = 0.16) and BMI (r = 0.34, p = 0.078). In the upper taxonomic levels, the genus *Collinsella* showed a positive significant correlation with triglycerides (r = 0.64, p = 0.00029) (Fig. 3c), and BMI (r = 0.39, p = 0.044) (Fig. 3d), and a negative correlation with HDL (r = − 0.4, p = 0.036) (Fig. 3e), and a positive but weak correlation with waist circumference (r = 0.35, p = 0.073). The same behavior was observed with the corresponding family Coriobacteriaceae, order Coriobacteriales and class Coriobacteriia (Additional file 5: Table S2). We also found positive correlations of tryglicerides and BMI with Clostridiales, Clostridia (Additional file 5: Table S2), and Firmicutes (Additional file 5: Table S2), which were more abundant in the OMS vs NW groups. Contrary, Bacteroidetes, Bacteroidia and Bacteroidales, which were over-abundant in NW when compared to OMS were negatively correlated with tryglicerides and BMI (Additional file 5: Table S2). In addition, *Faecalibacterium prausnitzii* (Fig. 3f) and *Faecalibacterium* (Fig. 3g) showed a positive correlation with BMI (r = 0.38, p = 0.048) while *Bifidobacterium adolescentis* correlated positively with triglycerides (r = 0.74, p = 0.0000094) (Fig. 3h) and a negative but weak correlation with HDL (r = − 0.34, p = 0.082). Both species were significantly more abundant in O with respect to NW. Furthermore, *Parabacteroides distasonis* which was more abundant in O when compared to OMS showed a positive correlation with LDL (r = 0.58, p = 0.0023) (Fig. 3i). Importantly, we found a positive correlation with waist-circumference and taxa in different levels, including Erysipelotrichi, Erysipelotrichales and Erysipelotrichaceae, and Bacilli (Additional file 5: Table S2), which were more abundant in OMS pairwise comparisons against NW and O.

### RNAseq resulted in a representative assembly of the metatranscriptome

**Fig. 4:**
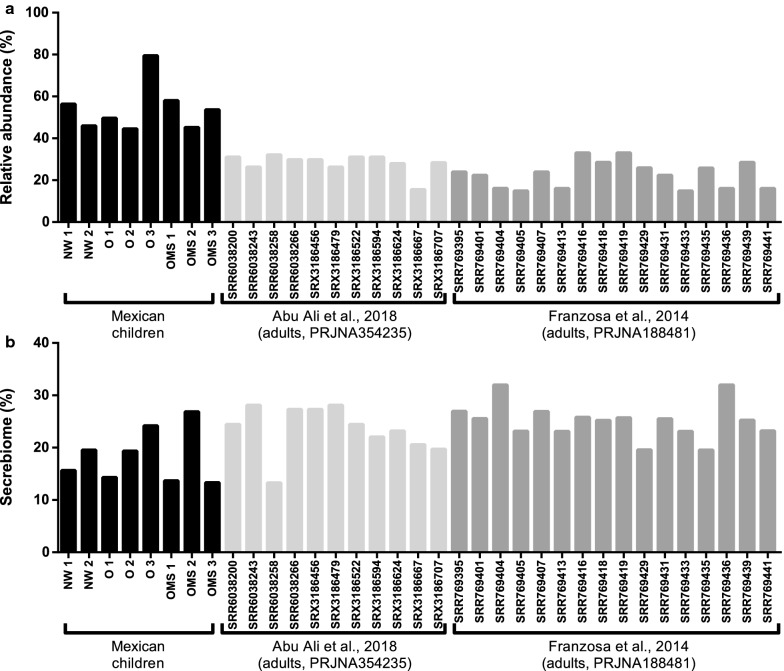
Remapped sequences to the metatranscriptome and Secrebiome. **a** Proportion of reads remapped to the global metatranscriptome and **b** proportion of Secrebiome/metatranscriptome remapped by sample. The external samples were taken from BioProjects PRJNA354235 and PRJNA188481

To explore the expressed taxonomy and functions associated with obesity and metabolic syndrome, we conducted a metatranscriptomic analysis. To this end, we obtained and sequenced the total RNA of eight fecal samples (NW = 2, OB = 3, and OMS = 3). The integrity of the total RNA showed the dominance of the 16S/23S and 18S/28S rRNA peaks (Additional file 6: Fig. S4). The samples were rRNA depleted and sequenced and after quality control and removal of sequencing artifacts, a total of 172,444,756 million reads with an average read length of 92 nt were obtained. After the removal of Eukaryotic and Prokaryotic ribosomal RNA and human transcripts, we obtained 110,014,240 RNA-seq reads, which were used for the de novo assembly of the metatranscriptome. Next, we obtained an assembly comprised of 224,427 contigs with an N50 of 702 bp and a total of 125,015,187 nucleotides assembled (Additional file 7: Table S3). From these contigs, we obtained 115,712 open reading frames (ORF) containing a protein sequence. Notably, > 54% of the total reads from the eight samples mapped back to the assembly, suggesting that our assembly covers a broad fraction of the sequence spectrum for all the samples (Fig. 4a). In the same manner, the read mapping to our assembly was performed with metatranscriptomic samples collected from twenty-seven healthy adults from two independent studies (NCBI BioProjects PRJNA354235 and PRJNA188481). Interestingly, > 25% of the reads mapped to our assembly (Fig. 4a), suggesting that our metatranscriptome also covered a good proportion of the sequence spectrum in adults, considering the known differences of the microbiota composition among children and adults.

### Determination of the Secrebiome

Proteins can be secreted by bacteria through multiple secretory mechanisms. Thus, we used a bioinformatic strategy [14, 15] to predict the secreted proteins encoded in our metatranscriptome. Out of the 115,712 genes that codify for proteins, we predicted 30,024 as potentially secreted, which represented ~ 26% of the total proteins in our metatranscriptome. All these proteins were referred to as the secretome of the microbiome, the “Secrebiome”. Notably, this represents 31.10% of the expression of the total metatranscriptome. From the 30,024 secreted proteins, 96.57% showed significant similarity against homologs in the NCBI’s non-redundant protein database (nr), 64.26% were assigned a Gene Ontology (GO) term, and 19.84% were assigned an Enzyme Commission (EC) number. The GO terms distribution showed that the Secrebiome was mainly composed of Hydrolase activity (18.4%) at Molecular Function (Additional file 8: Fig. S5a), membrane (37%) at Cellular Component (Additional file 8: Fig. S5b); and organic substance metabolic process (21%) at Biological process (Additional file 8: Fig. S5c). Finally, the EC classification showed that Hydrolases and Transferases were the most abundant terms with 47.5% and 23.4% of the EC sequences, respectively (Additional file 8: Fig. S5d). The GO and EC recruitment plots showed a relatively complete collection of the total ontology and enzyme variability as most curves passed their inflection points after 10,000 observations in most samples (Additional file 9: Fig. S6a, b), suggesting an adequate functional coverage of our samples to the Secrebiome.

Importantly, when we mapped the RNA-seq reads of the eight samples to the Secrebiome > 18% of the reads that mapped to the total metatranscriptome, aligned back to the Secrebiome (Fig. 4b), suggesting that an important proportion of reads potentially represented secreted proteins and thus may be in direct contact with the host. Notably, the reads from the two external published BioProjects, mapped back > 24% of the reads (Fig. 4b), suggesting that approximately a quarter of the sequences in the metatranscriptome of adults, correspond to the expression of the Secrebiome. Interestingly, the read proportion that mapped to our samples is more variable than the percentage obtained for the studies on adults, suggesting that the Secrebiome in children is more variable among individuals than in adults (Fig. 4b).

### Differential expression of the Secrebiome among obesity groups

**Fig. 5:**
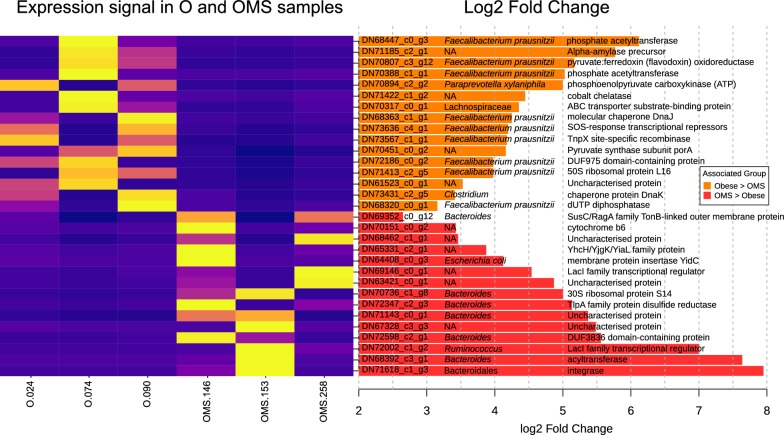
Differentially expressed secretome transcripts. Transcripts significantly associated with either the O or OMS were determined with DESeq after expression signal standardization. The heatmap shows the normalized RSEM abundance of each transcript per sample with a higher abundance in yellow. Bars on the right depict the total fold transcripts detected in association to each group (in log2 scale). The overlying matrix contains the predicted protein name on the left, the putative taxonomic assignation based on an LCA approach on the center, and the associated functional annotation on the right

A comparison of the predicted abundance values (RSEM) of the Secrebiome transcripts from the obese groups resulted in the identification of 31 transcripts differentially expressed in either the O (16 transcripts) and OMS (15 transcripts) groups (Fig. 5). Because RNA-seq data was only available for two samples in the NW group and their contribution to the set was limited, this group was excluded from the DESeq differential expression analysis to avoid a detrimental effect in the internal normalization. Regardless, the resulting 31 differentially expressed transcripts obtained for both the O and OMS groups were compared against an independent normalization carried out separately with the NW group to compare their expression in this group and the obese groups. As seen in Fig. 5 and Additional file 10: Figure S7, the fold change of expression of the 31 transcripts was mainly driven by a strong signal in the O and OMS groups, with a negligible impact from the NW group, suggesting that the comparison of the overexpressed genes is associated to the obesity phenotype.

The transcript DN71185_c2_g1, was one of the most strongly associated with the O group. It was cross-referenced to a carbohydrate-binding module (CBM26), and its sequence was homologous to an alpha-amylase precursor. Further, the taxonomy assigned to most of the differentially expressed transcripts corresponded to *Faecalibacterium* *prausnitzii,* mainly present in the O group (9 out of 16) and to the genera Bacteroides, mainly present in the OMS group (6 out of 15) (Fig. 5). Interestingly, nine of the 31 transcripts had significant correlations with anthropometric and biochemical measurements (Additional file 11: Fig. S8). The transcripts corresponding to a molecular chaperone DnaJ (DN68363_c1_g1) and a chaperone protein DnaK (DN73431_c2_g5) negatively correlated with LDL levels. One transcript corresponding to an integrase (DN71618_c1_g3) correlated positively with triglycerides and weight, and five transcripts correlated positively with diastolic and systolic blood pressure. Finally, two transcripts correlated negatively with glucose levels, TlpA family protein disulfide reductase (DN72347_c2_g3), and cytochrome b6 (DN70151_c0_g2). Furthermore, a gene coding for integrase (DN71618_c1_g3) was positively correlated with triglycerides and weight, while the 30S ribosomal protein S14 (DN70736_c1_g8) was also positively correlated with weight. All these transcripts can be suggested as potential biomarkers to differentiate the microbiome expression profiles among obesity and obesity with metabolic syndrome.

### CAZY enzymes were enriched in the Secrebiome

One of the most critical roles of the microbiota is its ability to utilize complex carbohydrate sources. Thus, classification according to the Carbohydrate-Active enzymes (CAZy) was performed to assess which expressed secreted proteins possessed the ability to enhance the carbohydrate-harvesting activity and analyze their association with obesity phenotypes. This analysis resulted in the identification of 2249 secreted proteins that had catalytic or carbohydrate-binding modules involved in the degradation and modification of carbohydrates. Interestingly, enrichment of CAZY enzymes was observed in the Secrebiome (7.5%) as compared to the non-secreted proteins (3.0%) of the metatranscriptome. The most abundant CAZY enzyme classes in the Secrebiome were Glycoside Hydrolases (GH; 35.1%), Carbohydrate-Binding Module (CBM; 33.8%), S-Layer Homology domain (SLH; 13.7%), and Carbohydrate Esterases (CE; 9.7%) (Additional file 12: Fig. S9).

**Fig. 6:**
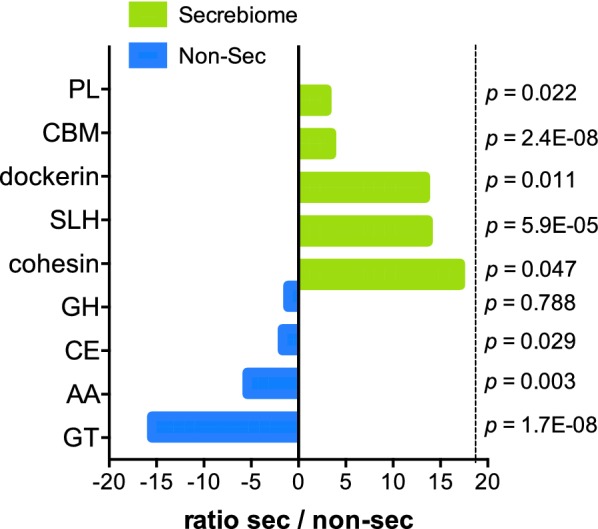
Ratio of Carbohydrate-Active enzymes (CAZys) in secreted and non-secreted proteins. The bars show the ratio of each CAZy family present in the total set of secreted (green)and non-secreted (blue) proteins. We used multiple t-tests to analyze the significant differences among the different enzyme families

We did not find significant differences in the distribution of CAZY enzymes within the Secrebiomes or within the non-secreted proteins of the three groups. However, when we compared the secreted against the non-secreted proteins, we found a significant abundance enrichment of CAZY enzymes from cohesin, SLH, dockerin, CBM, and Polysaccharide Lyases (PL) classes in the Secrebiome when compared to the non-secreted proteins of the metatranscriptome (Fig. 6). Contrastingly, a significant underrepresentation was observed in the Secrebiome for Glycosyltransferases (GT), Auxiliary Activities (AA), and CE CAZy classes. Only the abundance of the glycoside hydrolases was not significantly different among Secrebiome and non-secreted proteins (Fig. 6). These data suggest a differential CAZy activity in the Secrebiome. Interestingly, the families enriched in the Secrebiome seemed to be focused on binding and receptor functions, which suggest a role in the communication between the microbial communities and of the bacteria with the host.

### The Secrebiome of obesity had more abundance of antigenic regions (AAR)

**Fig. 7:**
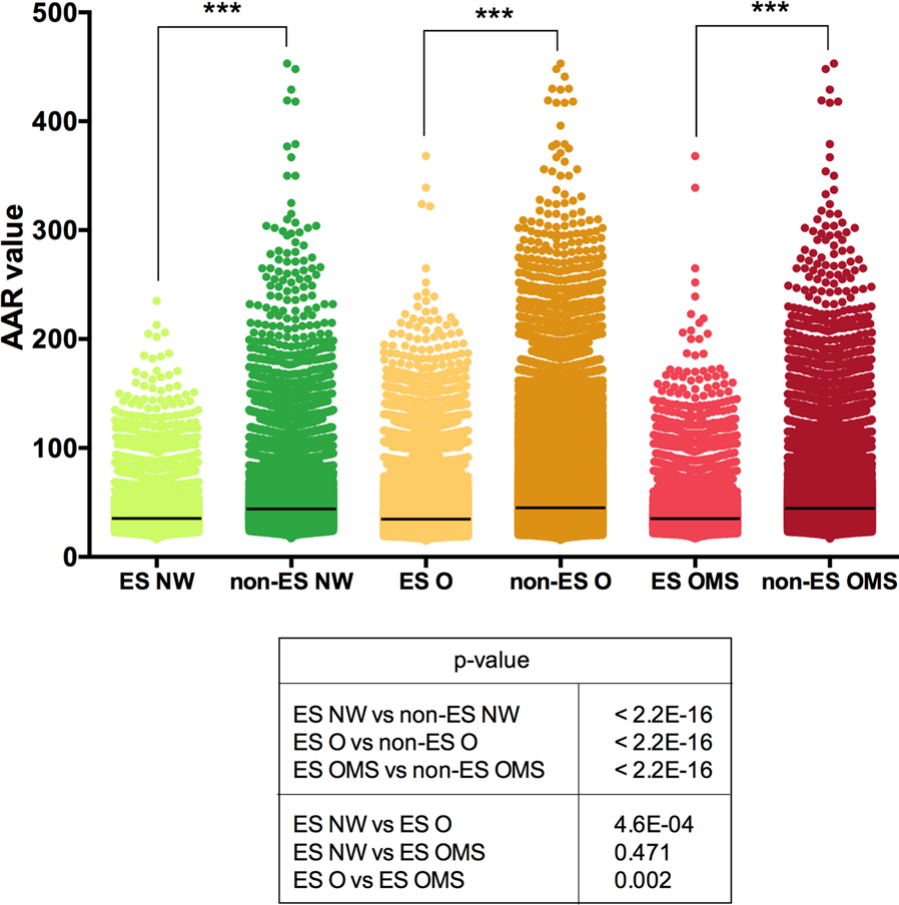
Comparison between the abundance of antigenic regions values (AAR) of secreted and non-secreted proteins in the different groups. The x-axis shows the protein set analyzed, and the Y-axis shows the AAR value obtained for each protein in the dataset. The line shows the mean value for each group. A Mann–Whitney test was performed to compare the AAR within each group with a confidence level of 99% (***p ≤ 0.001)

Because an important proportion of the expressed proteins may be secreted and therefore, be in direct contact with the host, we determined the antigenic potential of the Secrebiomes calculating the abundance of antigenic regions (AAR) value. We found that the Secrebiomes of NW, OB, and OMS had significantly more antigenic density (Mann–Whitney test p ≤ 0.0001) than the corresponding sets of non-secreted proteins for each phenotype (Fig. 7). Interestingly, the Secrebiome of the NW (AAR = 40.33) was more antigenic than OMS (AAR = 40.5) and O (AAR = 40.66). However, significant differences among Secrebiome was only observed in comparisons of NW vs O (p = 0.0004673), and O vs OMS (p = 0.002006), while NW vs OMS was not significant (p = 0.4709). This result suggests that the Secrebiome associated with obesity decreased the expression of proteins associated with a higher antigenic density.

## Discussion

The current study presents, to the best of our knowledge, the first insight into the expressed genes corresponding to the secreted proteins of the microbiome, the Secrebiome, associated with obesity with, and without metabolic syndrome, in children around 9 years of age. The current Mexican cohort reflects the unrelenting prevalence of the obese population worldwide, a growing problem that is more pronounced in developing and developed countries. In 2017, the Organization for Economic Cooperation and Development (OECD) reported that the United States of America and Mexico led obesity rankings. In these terms, children are a particularly susceptible population that has been often overlooked by governmental policies and scientific studies but comprise an essential economic and health issue for the future of these countries. In our cohort of 750 individuals, we found that 52.94% of the obese children have metabolic syndrome, which is similar to the 62% obtained in previous reports for Mexican obese children [24].

The main focus of the current study was to assess the actual expressed profiles of the microbiota, using a metatranscriptomic approach (RNA-Seq). Whereas the metagenome may show the full functional potential of the microbiota, only a small fraction of the bacterial genes are expressed [25]. Thus, a metatranscriptomic approach provides a more accurate description of the underlying dynamics of obesity, by capturing the community transcript population representing each taxonomic group in the study [25]. Although our results from metatranscriptomics should be interpreted with caution due to the limited sample size, this work provides a framework for further studies using metatranscriptomics to analyze the expressed genes of the Secrebiome and its close relationship with the host under diseases.

We used the V4 region of the 16S rRNA gene to establish the taxonomic profile of the microbiota from fecal samples of three children groups: normal weight (NW), obese (O), and obese with metabolic syndrome (OMS). We observed that the three groups are dominated by the same three phyla, Firmicutes, Bacteroidetes, and Proteobacteria. However, we detected specific changes in the microbiota structure depending on the obesity type. The OMS patients had the highest bacterial diversity, followed by O and NW patients. This observation is in agreement with other studies with big cohorts of obese patients that also observe an increase in diversity in them [26]. Contrary, some studies revealed a decrease in diversity in obese patients [27, 28]. Regarding the Mexican children population, one study shows a higher diversity associated with obesity [29], and the other two studies showed no difference in diversity among obesity and normal-weight controls [30, 31]. Thus, the role of bacterial richness and diversity in children obesity remains unclear, suggesting that more studies are necessary. The PCoA analysis of the microbiota showed that the most compact cluster was formed among the NW samples, while the most disperse was the OMS group; the O group seemed to be an intermediate state of healthy and obese with metabolic syndrome. Similar clustering behavior was recently observed as a consequence of metabolic abnormalities in the adult population [26].

After analyzing the differentially abundant taxa, we observed that the genus *Porphyromonas* and an undetermined species within this genus were specifically over-abundant in the O group, suggesting that these taxa could be potentially used as biomarkers of obesity. Although we did not evaluate a concrete species, the presence of a typical member of this genus, *P. gingivalis* has been associated as a risk factor for developing obesity and diabetes [32]. Interestingly, we found that the class Coriobacteria, order Coriobacteriales, family Coriobacteraceae, genus *Collinsella* and species *Collinsella aerofaciens* were significantly more abundant, specifically in the OMS as compared to O and NW (Fig. 2), suggesting them as potential biomarkers for obesity with metabolic syndrome. Furthermore, this species also showed a significant positive correlation with triglycerides (r = 0.62, p = 0.00053), while showing a negative correlation with HDL (r = − 0.4, p = 0.039). Also, the genus *Collinsella* correlated positively with triglycerides (r = 0.64, p = 0.00029), BMI (r = 0.39, p = 0.044), and a negative correlation with HDL (r = − 0.4, p = 0.036), and had a weak positive correlation with waist circumference (r = 0.35, p = 0.073). The same behavior was observed with the corresponding family Coriobacteriaceae, order Coriobacteriales and class Coriobacteriia (Additional file 5: Table S2). We suggest that these taxa may be considered as biomarkers for OMS. In this regard, it was recently reported that an altered abundance of *Collinsella* genus changes the host’s plasma cholesterol levels, showing a positive correlation with LDL cholesterol [33]. Additionally, another study with big cohorts of adults recently reported the finding of an increased *Collinsella* abundance in obese patients [26]. Also, it has been observed that low dietary fiber intake increases *Collinsella* abundance in the gut microbiota of obese pregnant women [34]. Evidence from literature strongly suggests that Coriobacteraceae are important constituents of gut microbiomes affecting the physiology of human and mice hosts [35]. Thus, it appears crucial to evaluate host-microbe interactions in finer detail, in the context of host lipid and cholesterol metabolism and their role with obesity and obesity with metabolic syndrome.

Importantly, the abundance of *Parabacteroides distasonis* was significantly decreased in OMS as compared to O but not with respect to NW. Interestingly this species correlated positively with LDL cholesterol levels (r = 0.58, p = 0.0023). Recently, a study showed that *P. distasonis* produces metabolites that can reduce weight gain and hyperglycemia as well as improve glucose metabolism and symptoms of obesity-related conditions such as liver disease [36]. In this regard, our findings highlight the important effects of this species as a biomarker of obese children with metabolic syndrome. Also, the class Erysipelotrichi, order Erysipelotrichales, family Erysipelotrichaceae, genus *Catenibacterium* and an undetermined *Catenibacterium* species were significantly more abundant in OMS groups when compared to the O and NW, and may prove interesting biomarkers for obesity with metabolic syndrome. This genus has only one species that has been properly described (*Catenibacterium mitsuokai*), and a study with humanized mice fed with a “western” diet revealed an increased representation of *Catenibacterium mitsuokai*, in the fecal samples of these animals when compared with mice fed with a low fat/plant polysaccharide diet [37].

There is evidence from literature documenting a potential role for the family Erysipelotrichaceae in host physiology. The increased abundance of this family has been associated with host dyslipidemia in the context of obesity, metabolic syndrome and hypercholesterolemia [38–40]. Additionally, nutritional studies also support the influence of dietary fat on the abundance of this family [41]. Our data highlights the importance of the family Erysipelotrichaceae in metabolic syndrome of obese children and suggest that *Catenibacterium* and its undetermined species may be interesting as novel biomarkers of this disease. On the other hand, we also found an increased abundance of Bacteroidales in NW as compared to the OMS group, and an increase in the abundance of Clostridiales in OMS as compared to NW group. Recently, a report in adults determined that the microbial gut community of obese people was characterized by higher Clostridiales whereas lean people tend to have higher Bacteroidales counts [42]. The microbiota of obese adults recently revealed a high abundance of *Coprococcus*, a genus of Clostridiales [43], as well as in pregestational obesity [44], which is in accordance with our data, suggesting that these bacteria are important determinants in children obesity with metabolic syndrome.

The N50 of our metatranscriptome assembly was ~ 700 nt, which is congruent to the expected length of Prokaryotic mRNA molecules, (~ 900 pb long). Additionally, > 54% of the total reads from our samples mapped back to the assembly, suggesting an adequate assembly, even better than the ones obtained in other studies using a similar methodology [45]. Indeed, when we remapped sequencing data from different cohorts, we recovered ~ 25% of the reads despite them being of healthy adults from different populations, so this metatranscriptome and Secrebiome could be considered as a reference for further studies in different populations. Interestingly, the read proportion that mapped back to the Secrebiome from our children samples tended to be lower and more variable than the read proportion of the adult studies, suggesting that the adults tend to maintain a stable core of the Secrebiome, while children are more variable in read mapping abundance.

Bioinformatic prediction of the expressed genes corresponding to the Secrebiome is a novel methodology that could help us discover and understand novel communication mechanisms between the host and the microbiota. We found that ~ 26% of the total metatranscriptome expressed genes on the gut microbiota can be flagged as secreted proteins. The study of CAZymes in obesity studies is crucial due to their role in the degradation of complex carbohydrates that can be absorbed by the intestinal colonocyte. Contrary to a previous study of CAZymes in metagenome of obesity [46], we did not find differences in the Secrebiome between NW, O, and OMS, possibly due to the experimental design of our study. Still, we found that the abundance of *Faecalibacterium* and *Faecalibacterium prausnitzii* were significantly increased in O as compared to NW (Fig. 1e) and that this species had a significant positive correlation with BMI (r = 0.38, p = 0.048). It is well documented that *F. prausnutzii* can act as a probiotic due to its production of butyrate [47, 48], and it has been reported as depleted from the gut microbiota in individuals with metabolic syndrome [49], although we did not find a total depletion of this species in OMS group. However, a recent study with adult cohorts of obese patients found an increased abundance of *Faecalibacterium* [26], and also in a Mexican obese children cohort, an increase of abundance was reported [30]. Thus, further studies are necessary to clarify the role of *Faecalibacterium* in obesity.

Secreted proteins vary among the bacterial realm, as secretion systems are widely variable in complex communities such as the gut [50]. After establishing the characterization of the secreted proteins in our samples, the Gene Ontology and Enzyme Commission numbers showed a substantial prevalence of surface and membrane proteins in the Secrebiome. Furthermore, we also detected a marked prevalence of catabolic enzymatic activity and binding proteins that may contribute to nutrient uptake, polymer biodegradation, or cell attachment to the substratum or other cells. The Secrebiome presented a very different CAZY profile when compared to the rest of the metatranscriptome, with the Secrebiome being enriched in carbohydrate-catabolism proteins, namely glycoside hydrolases, carbohydrate-binding module, SLH domain bearing proteins, and carbohydrate esterases. Although the corresponding CAZy distribution was not group-associated, as a whole, the Secrebiome was particularly enriched in the production of SLH, and the dockerin-cohesin complex, congruent to binding requirements for the assembly of cellulosomes. Additionally, the abundance of antigenic regions (AAR), which evaluates antigenic density, found differences that were statistically significant between the NW and the O groups. As previous studies by our group have shown, the secretomes of more virulent pathogenic bacteria tend to have a higher antigenic density; this may be an indicator of pathogen activity in the obese subjects that may deserve further study [15, 51].

A total of 31 specific transcripts within the Secrebiome were deemed differentially abundant in association with either the O or OMS group. Since most were virtually absent from the NW (Fig. 4), we infer they may be related to the subjects’ condition. Interestingly, most of those presenting a high fold change in the O group (Fig. 5) were homologous to the genome of *Faecalibacterium prausnitzii,* which, as mentioned previously, has been considered as probiotic. Contrastingly, most differentially expressed transcripts associated with the OMS group were homologous to the pangenome of *Bacteroides*, a genus that has been commonly reported to be associated with overweight subjects [52]. *Bacteroides* species were also predominant among urban gut microbiomes and contained unique gene clusters, which encode different CAZymes, whose functions could be related to an energy over-extraction of the microbiota form OMS patients [53]. Even so, it is essential to note that although we can evaluate the taxonomy of these specific marker transcripts, this is by no means a thorough representation of the whole taxonomy and cannot rule out their presence and activity in the O or NW groups. Only some correlations were significant with the clinical data associated with obesity. Of these, perhaps the most interesting were those with LDL, commonly regarded as “bad cholesterol”. Two chaperone proteins were found to be negatively correlated to LDL mg/dL, DnaK (HSP70), and DnaJ (HSP40), both more prevalent in the O group and less in the OMS group. These have been reported to interact with one another to carry out ATP-hydrolysis, which may be related to the thermogenesis and binding with unfolded polypeptide chains to prevent their aggregation.

Overall, this study showed that the definition of the Secrebiome provided new information on the gut microbiome functions that are directly related to the host communication, helping us to understand the functional interplay within the holobiont and open new insights in the study of the expressed microbiome. These findings may provide valuable insights to understand how the expressed bacterial genes and their respective proteins in the gut influence the metabolic response of the host to different nutrients and the alterations during obesity and obesity with metabolic syndrome.

## Conclusions

One of the most exciting roles of the microbiota is its capacity to interact with the host and influence the overall health state. One of the mechanisms that the bacteria use to this end, is the secretion of proteins that interact directly with the host, metabolizing the nutrients for energy harvest and binding to host-cell receptors for signaling activities. The 16S rRNA profile of our samples showed the enrichment of taxa that have been previously reported as biomarkers for obesity such as Coriobacteraceae and *Collinsella* and suggest some new ones to be considered such as an increase of *Collinsella aerofaciens* and Erysipelotrichaceae, *Catenibacterium* and *Catenibacterium sp.*, and a decrease of *Parabacteroides distasonis*, which correlated with clinical and anthropometric parameters of obesity and metabolic syndrome. After comparing our metatranscriptome and Secrebiome with data from studies of other populations we observed that the proportion of mapped sequences was more variable for children samples than for adults, suggesting the establishment of a core Secrebiome in the adult population; further, given the high proportion of sequences that mapped back to our assembly, it could be considered as a reference for further studies in different populations. The differential expression analysis of the genes of Secrebiome proteins showed 31 significantly overexpressed genes for the O and the OMS group. Interestingly, these genes were homologous to *F. prausnutzii* in the O group and Bacteroides in the OMS group. Although this data does not establish the causal role of these taxa to the disease phenotypes, it cannot be discarded that some taxa could cause the dysbiosis present in a disease state.

Finally, the analysis of CAZy enzymes showed a differential distribution between the secreted and non-secreted proteins. The Secrebiome showed an increased presence of cohesin, SLH, dockerin, CBM, and PL domain bearing proteins, showing a potential critical role of the secreted proteins in the degradation and utilization of carbohydrates as nutrients. Overall, this study showed that the definition of the expressed genes of the Secrebiome provided new information on the gut microbiome functions that are directly related to the host health state and provide essential insights to understand how the bacterial proteins in the gut could influence the metabolic response of the host to different nutrients. The metatranscriptome and 16S profiling demonstrated the importance of the separation of obesity from obesity with metabolic syndrome patients for a better understanding of the microbiome role in the disease.

## Methods

### Study population, anthropometric and clinical parameters

We analyzed the stools from 10 normal weight (NW), 10 obese (O), and 7 obese with metabolic syndrome (OMS) children, aged 7–10 years old, from a summer-camp of children of Mexican Health Ministry employees. All children came from households with a middle economic class income and belonged to a similar socio-cultural status. All of them lived in Mexico City at the time of collection and did not practice any sport regularly. The study groups were paired by gender and age. Samples were refrigerated at home at 4 °C and transported to the research facility within the following 12 h after collection in a portable cooler with ice packs to preserve the temperature. All samples were received at the research facility in the early morning; 200-mg aliquots were made and stored at − 70 °C in sterile plastic containers with RNA later. Obesity was defined by body mass index (BMI) ≥ 95th percentile, whereas NW was defined as BMI between the 15th and 75th percentiles for age and gender, based on the guidelines of the Centers for Disease Control and Prevention (CDC). Metabolic syndrome parameters were determined according to previous reports [24], and OMS were defined by the presence of waist circumference > 75th by age and gender, and at least two of the following metabolic traits: (1) triglycerides > 1.1 mmol/L (100 mg/dL); (2) HDL cholesterol < 1.3 mmol/L (50 mg/dL), (3) glucose > 6.1 mmol/L (110 mg/dL) and (4) systolic blood pressure > 90th percentile for gender, age, and height. Blood samples of 5 mL were drawn after 8–12 h of fasting on the same day of the feces collection. Children in the O group were selected so that they did not have more than one trait matching the metabolic syndrome traits. Exclusion criteria for all samples included recent bodyweight loss > 10%, antibiotic intake 3 months before sample collection, and the occurrence of diarrhea or acute gastrointestinal illness during the same period. The Ethics Committee approved the study of the Instituto Nacional de Medicina Genómica (INMEGEN) in Mexico City, Mexico. The parents or legal guardians of each child signed the informed consent form for participation, and all children assented to participate. Anthropometric parameters (Additional file 1: Table S1), blood pressure, and body mass index were measured following standardized procedures, as previously described.

### Sample collection and DNA/RNA extraction

Total bacterial metagenomic DNA for 16S rRNA amplicon sequencing was extracted from 200 mg of feces using the QIAamp^®^ DNA Stool Mini Kit (Qiagen, Inc.; Hilden, Germany) following the manufacturer’s protocol. The total RNA extraction was performed with a combination of the ZR Soil/Fecal RNA MicroPrep (Zymo Research; California, USA) and the RNeasy Mini Kit (Qiagen, Inc.; Hilden, Germany), according to manufacturer’s protocol. The total RNA quality was assessed with an Agilent 2100 bioanalyzer and quantified with a Qubit 2.0 Fluorometer. Human and bacterial ribosomal RNA was removed using Ribo-Zero Gold rRNA Removal Kit (Illumina; California, USA) following manufacturer instructions.

### High-throughput 16S rRNA profiling and RNA-Seq

The V4 hypervariable region was amplified using 515F and 806R primers following the protocol by Caporaso and collaborators [54]. The amplicons were prepared using 100 ng of total DNA, and products were confirmed by agarose gel and purified using Agencourt AMPure XP beads (Beckman Coulter). Fragment size and DNA concentration of each amplicon were determined using an Agilent D1000 ScreenTape for 4200 TapeStation System (Agilent Technologies) and a Qubit 2.0 fluorometer (Invitrogen), respectively. Amplicons were sequenced using an Illumina MiSeq platform at the INMEGEN using reagents for 2 × 250 paired-end sequencing.

The RNA-seq libraries were prepared using the NEBNext Ultra RNA Library Prep Kit for Illumina (New England Biolabs; Massachusetts, USA). In brief, total RNA previously depleted of rRNA was fragmented at 94 °C for 14 min, followed by first-strand retrotranscription and second cDNA synthesis with random primers using Klenow fragments. Next, we performed the ligation of Illumina compatible indices for multiplex after repairing DNA ends and adding a dA-tail to each strand. Finally, we performed the enrichment PCR amplification with 12 cycles following the manufacturer’s instructions. The quality and quantity of the resulting libraries were assessed with a Qubit 2.0 Fluorometer and Agilent 2100 bioanalyzer. All RNAseq libraries were sequenced at the INMEGEN sequencing service using the Illumina NextSeq500 platform for 2 × 150 paired-end sequencing.

### Bioinformatic analysis of the 16S rRNA profiling data

We applied a quality filtering (> Q20 Phred score) and ambiguous nucleotides removal. The resulting reads were joined and analyzed using the QIIME 1.9.1 package. Amplicon sequences were clustered at 97% identity into operational taxonomic units (OTUs) guided by the Greengenes Database (version 13_8) using UCLUST allowing for reverse strand matches following a closed reference-based clustering approach. We assigned the taxonomy to the resulting Operational Taxonomic Unit (henceforth OTUs) based on the one from 97% identity clusters of the Greengenes database. Sequences not aligning the references were not considered for downstream analyses. We eliminated the OTUs accounting for ≤ 0.005% of the total read abundance (80 cumulative reads) from downstream analyses. A valid taxonomy was assigned to the reads, and it was collated and reported in terms of relative abundance. UniFrac distances were calculated with scripts from the QIIME v1.9 suite. Data ordination was carried out from the distance matrices using a principal coordinate analysis (PCoA) with vegan using in-house scripts. Differentially abundant taxa (group-specific) were identified with the LDA Effect Size algorithm (LEfSe Galaxy Version 1.0) using a standardized taxonomy table (mean of 10,000 rarefactions at a depth of the smallest sample) to cope with uneven sample size. No per-sample normalization of the sum of the values to 1 M was used, and a minimum LDA score of 1 was considered. Group pairwise comparisons were carried out as well (not group-specific).

### Bioinformatic analysis of RNA-Seq data

The process is briefly described as follows. The first step was the quality assessment by FastQC (Andrews S. (2010). We filtered using a 20 Phred quality score trimming with Trimmomatic v 0.36 with a sliding window of 6 nt, following the removal of sequencing adapters and ambiguous bases. The resulting high-quality sequences were depleted of Prokaryotic and Eukaryotic rRNAs using Ribopicker v.0.4.3 and the SILVA rRNA database (132 release). The remaining non-rRNA sequences were aligned to the human genome and transcriptome (GRCh38_snp_tran) using HISAT2. Human-filtered sequences were then used as the input for the de novo transcriptome assembly with the Trinity metatranscriptomic assembler. After that, the original reads were aligned to the transcriptome using Bowtie2 v2.3 as part of the Trinity pipeline. The abundance of expression was determined by FPKM for the normalization of sequencing depth and transcripts length, considering only the transcripts with FPKM > 1. TransDecoder (https://github.com/TransDecoder/) was used to identify the longest ORFs candidate of protein coding regions within transcript sequences. The resulted protein sequences were annotated with hmmscan from the HMMER suite v3.1 against the dbCAN to obtain the carbohydrate-active enzymes (CAZY). Protein sequence homology was annotated by BLASTP against the NCBI non-redundant (NR) and functionally mapped to Gene Ontology (GO) terms using Blast2GO. The E-value cut-off was set at 1.0E−3.

### Secrebiome definition

To assess the set of secreted proteins of the metatranscriptome, we followed the bioinformatic strategy previously published in Cornejo-Granados et al. 2017. Briefly, the complete set of proteins was analyzed independently with six different feature-based tools to identify the excreted/secreted proteins (ES) by different secretion pathways and removing the ones that had transmembrane domains. All secreted proteins were analyzed with BLASTP against the NCBI’s non-redundant (nr) database using Blast2GO with an E-value cut-off set at 1.0E−3 to identify homolog proteins. Additionally, all proteins were functionally mapped to GO terms and annotated by setting the following parameters: E-value-hi-filter: 1.0E−3; Annotation cut-off: 55; GO weight: 5 and Hsp-Hit Coverage cut-off: 0. Finally, we used Blast2GO to identify the over or under-represented GO and EC terms in the ES proteins, by setting the term filter *p* value to ≤ 0.05.

### Differential gene expression analysis

We selected the genes of the Secrebiome with an FPKM > 1 for the differential gene expression analysis. In this manner, a gene is only considered if it is covered by reads in 1 kB long. These cut-offs were more stringent than those used in previous studies. Protein isomeres detected in the secretome were subjected to filtering for reducing data sparsity. The expression measurement was in RNA-Seq by Expectation–Maximization (RSEM), a reference-free transcript quantification method. Briefly, the expression below 1 in any sample was not considered, and isomeres with less than 2 cumulative observations across the samples, or those appearing in less than 3 samples or less than 2 groups were discarded. The RSEM of the resulting core isomeres in the O and OMS groups were standardized with the Differential expression of RNA-Seq (DESeq 2 v. 1.26.0) R package using default parameters, and those that were more abundant in either group were subsequently identified. Isomeres with a p-value below an α of 0.05 were considered as differentially abundant expression markers (given by log2 fold change) and were used for downstream analyses. Identified gene markers associated with either the O or the OMS groups were compared with the NW samples by standardizing the RSEM tables with DESeq using the three groups. Pearson/Spearman’s rank correlations between the gene markers and clinical data were calculated with in-house scripts. Cross-referenced GO and EC tables were filtered to retain only proteins identified with a unique identifier. These were collated per sample and the tables were standardized following the same procedures for the protein isomeres. These were then used for the calculation of recruitment plots using vegan with in-house scripts. The sequences corresponding to the differentially abundant transcripts identified with DESeq were used for homology search against NCBI’s NT database using the blastn algorithm from the BLAST + suite v 2.10 using with expected value 0.0001, id = 0.80, coverage 75%, Match/Mismatch 2,-3, and Gap/ext 5,2. If trailing hsps had a difference of > 7.5% identity, only the former was considered, and hits with no taxonomic information were ignored. The associated taxonomy was determined with a last common ancestor approach of all results.

### Search of CAZymes in the metatranscriptome and Secrebiome

We downloaded the CAZy database from http://bcb.unl.edu/dbCAN2/download/Databases/dbCAN-old@UGA/ containing 921,174 sequences as of September 2017. The detection of Carbohydrate-Active enzymes was performed using HMMER v3.1. Briefly, we prepared the CAZy HMM database with hmmpress using default parameters, and searches were carried out with hmmscan using the predicted proteins of the metatranscriptome and Secrebiome as inputs. Finally, we parsed the results using hmmscan-parser.sh and in-house scripts.

### Calculation of the abundance of antigenic regions (AAR)

The AAR is a value used to estimate the antigenic density of a protein, calculating the number of antigenic regions and normalized by the sequence length. We calculated this value using the Secret-AAR webserver for the different protein data sets [51]. Then, we used a Mann–Whitney test (p < 0.001) to establish if there was a significant difference between AAR values of the different data sets.

### Data accessibility

The sequencing data have been deposited in the NCBI GEO repository and can be consulted under the accession number GSE143207. Requests for additional material should be made to the corresponding author.

## Supplementary information

**Additional file 1: Table S1.** Anthropometric and biochemical characteristics of the analyzed population.

**Additional file 2: Figure S1.** Alpha diversity per sample at max depth; (a) Shannon index and (b) Observed OTUs.

**Additional file 3: Figure S2.** Top ten most abundant taxa: Per group a) phyla, b) families and c) genera per group; and per sample d) phyla, e) families and f) genera. NW = Normal Weight, O = Obese, and OMS = Obese with Metabolic Syndrome.

**Additional file 4: Figure S3.** Principal coordinate analyses of Weighted UniFrac distances. Distances are based on taxa abundance. The two linear combinations explaining the most variation are shown as dimensions 1 and 2. Elipses were calculated based on the most distant samples per group. Samples for which RNA-seq information is available are presented with a larger font.

**Additional file 5: Table S2.** Pearson correlations between clinical/anthropometric parameters and significantly abuindant taxa among NW, O, and OMS.

**Additional file 6: Figure S4.** Bioanalyzer profile of each sample used for the metatranscriptome.

**Additional file 7: Table S3.** Basic statistics of the metatranscriptome assembly.

**Additional file 8: Figure S5.** GO terms and Enzyme commission class distribution of the total ES proteins. Each pay graph shows the different GO and enzyme terms associated with the complete ES proteins encoded in the metatranscriptome for a) Molecular Function, b) Cellular Component, c) Biological Process, and d) Enzyme categories.

**Additional file 9: Figure S6.** Recruitment of unique functional categories per sample. Samples are colored by NW, O, and OMS groups. a) Recruitment of unique Gene Ontology (GO) features in progressive randomized no-replacement rarefactions based on Hurlbert calculations. b) Recruitment of unique Enzyme Commission numbers (EC) in progressive randomized no-replacement rarefactions based on Hurlbert calculations.

**Additional file 10: Figure S7.** Per-sample expression of transcripts associated with the O and OMS groups. The total normalized RSEM abundance of each transcript per sample with a higher abundance in yellow. Only differentially abundant transcripts associated with the case groups are shown. DESeq-based standardization of the expression signal was carried out considering all samples.

**Additional file 11: Figure S8.** Correlations between differentially abundant transcripts and clinical data. Only significant correlations are shown (α = 0.05). Pearson correlation, the p-value is shown. A linear regression model is fitted to the data using the transcript as a predictor; the intercept and slope are shown, and SE is presented as a shadow. Transcripts (x-axis) are shown as relative normalized RSEM abundances. The scale of the clinical data is different for each parameter. **a–d)** Transcripts positively correlating with diastolic blood pressure (percentile). **e–f)** Transcripts negatively correlating with glucose levels (mg/dL). **g–h)** Transcripts negatively correlating with LDL levels (mg/dL). **i–l)** Transcripts positively correlating with systolic blood pressure (percentile). **m)** Transcripts positively correlated to triglyceride levels (mg/dL). **n–o)** Transcripts positively correlated to subject weight (g).

**Additional file 12: Figure S9.** Carbohydrate-active enzyme distribution. Relative abundance of CAZy enzyme families across the secreted and non-secreted proteins in the NW, O, and OMS groups.

## Data Availability

The sequencing data have been deposited in the NCBI GEO repository and can be consulted under the accession number GSE143207. Requests for additional material should be made to the corresponding author.
