# Supplementary material for: Metatranscriptomic analysis to define the Secrebiome, and 16S rRNA profiling of the gut microbiome in obesity and metabolic syndrome of Mexican children
Source: Microb Cell Fact. 2020 Mar 6;19:61. doi: 10.1186/s12934-020-01319-y (PMC7060530; doi:10.1186/s12934-020-01319-y)
Supplement: Supplementary file 1 — Additional file 1: Table S1. Anthropometric and biochemical characteristics of the analyzed population. [file 12934_2020_1319_MOESM1_ESM.pdf]

Table S1. Anthropometric and biochemical characteristics of the analyzed population

**Characteristics**	**Group NW**	**Group O**	**Group OMS**	**P-value**
**Sample number**	10	10	7	
**Age** (year)	8.59±0.95	9.01±0.85	8.99±1.08	0.6752
**Gender** (male/female)	6/4	7/3	5/5	0.8566
**BMI** (kg/m2)*	39.3±16.98	96.2±1.47	98±1.31	< 0.0001
**WL** (cm)*	58.32±3.67	82.44±9.4	89.19±7.45	< 0.0001
**SBP** (mmHg)*	28.9±10.85	46.8±30.46	51.57±30.25	0.353
**DBP** (mmHg)*	70.90±10.53	59.3±20.52	70.86±19.12	0.3983
**GLU** (mmol/L)*	86.20±5.34	91.60±2.73	86.43±4.66	0.1103
**Blood lipid**				
**TC** (mmol/L)*	172.72±19.22	178.7±26.85	174.08±26.59	0.7477
**TG** (mmol/L)*	56.6±19.47	80±27.05	185.57±84.83	0.0004
**LDL** (mmol/L)*	98.90±20.02	115.7±28.36	109.8±19.87	<0.00X
**HDL** (mmol/L)*	62.5±10.82	47±8.5	37.8±6.68	0.3661

*__BMI:__ Body mass index; **WL:** Waistline; **SBP:** Systolic blood pressure; **DBP:** Diastolic blood pressure; **GLU:** Blood glucose; **TC:** Total cholesterol; **TG:** Triglyceride; **LDL:** Low density lipoprotein; **HDL:** High density lipoprotein;

#: Kruskal-Wallis test.
