# Supplementary figures and images for: Metatranscriptomic analysis to define the Secrebiome, and 16S rRNA profiling of the gut microbiome in obesity and metabolic syndrome of Mexican children
Source: Microb Cell Fact. 2020 Mar 6;19:61. doi: 10.1186/s12934-020-01319-y (PMC7060530; doi:10.1186/s12934-020-01319-y)

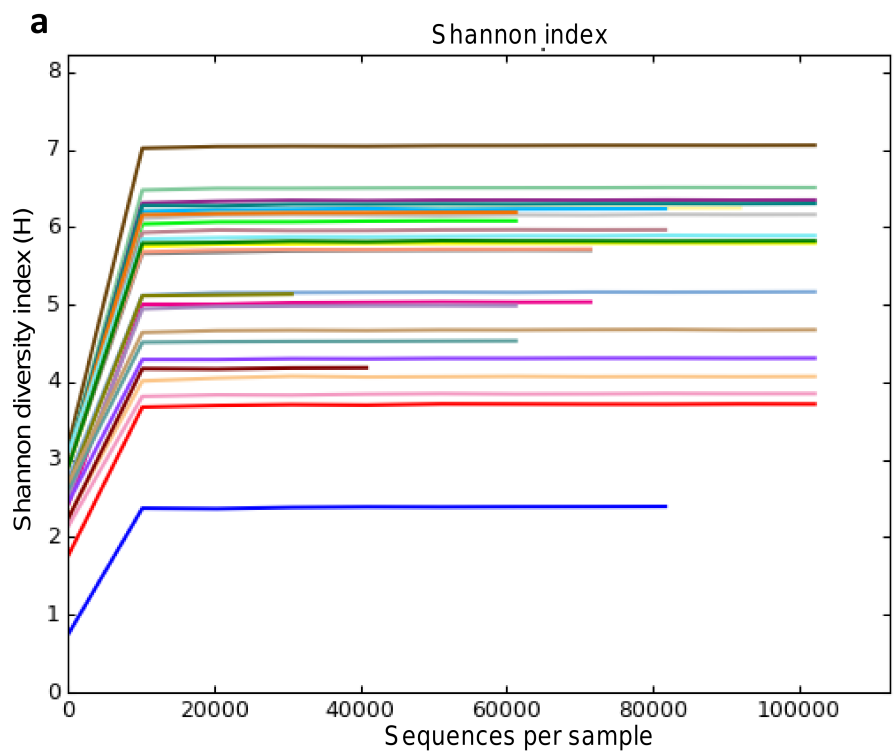

Legend

|     |
|-----|
| 010 |
| 015 |
| 024 |
| 039 |
| 064 |
| 074 |
| 087 |
| 124 |
| 146 |
| 147 |
| 152 |
| 153 |
| 161 |
| 164 |
| 165 |
| 169 |
| 193 |
| 197 |
| 258 |
| 288 |
| 314 |
| 418 |
| 420 |
| 434 |
| 445 |
| 446 |
| 090 |

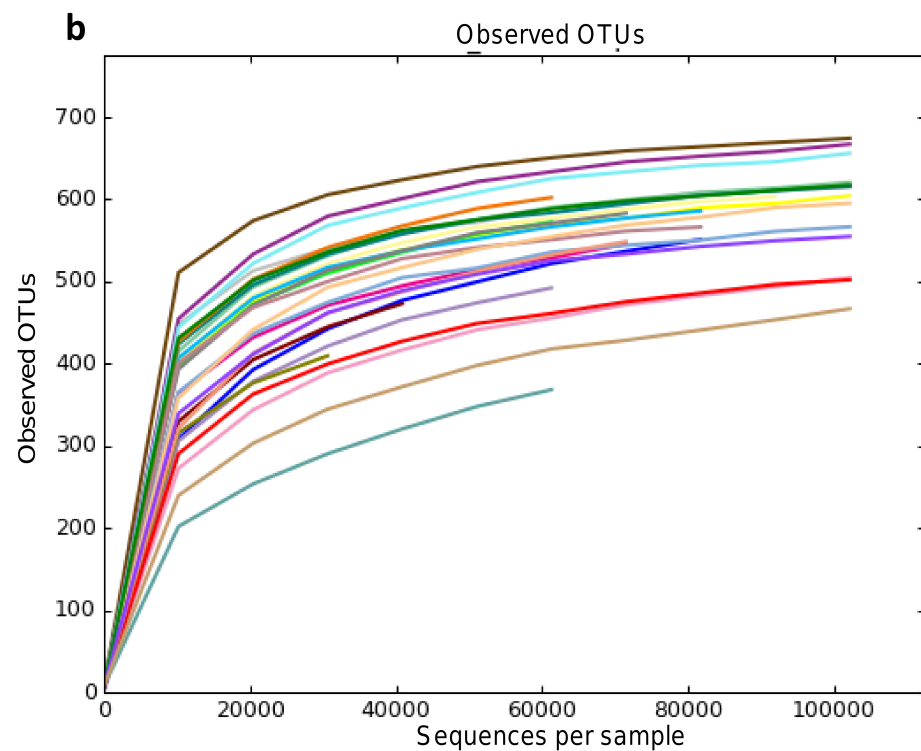

Supplement: Supplementary file 2 — Additional file 2: Figure S1. Alpha diversity per sample at max depth; (a) Shannon index and (b) Observed OTUs. [file 12934_2020_1319_MOESM2_ESM.pdf]

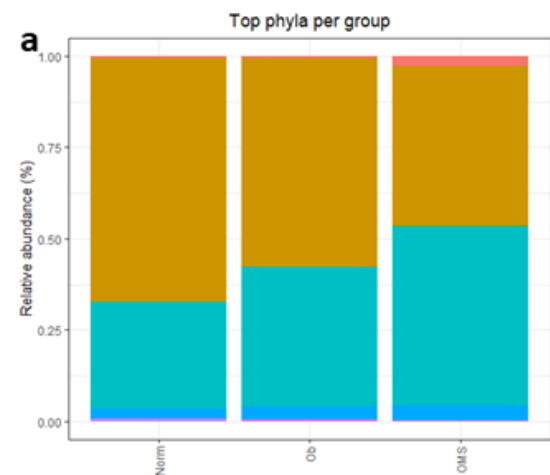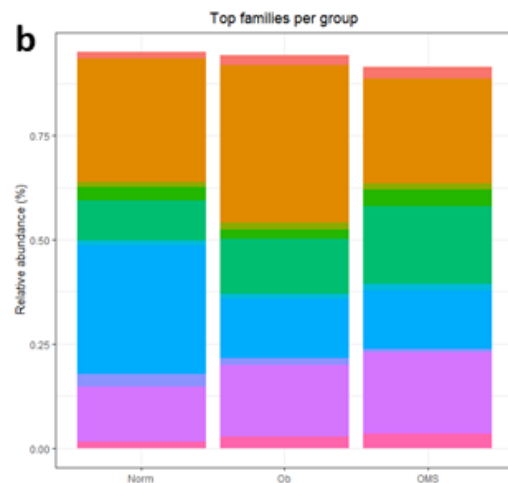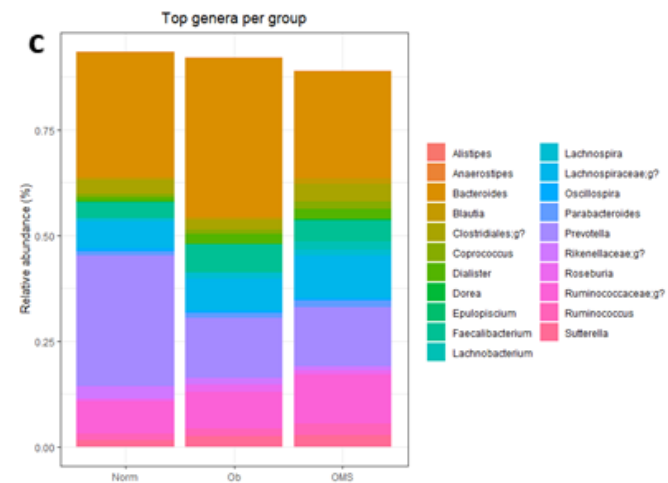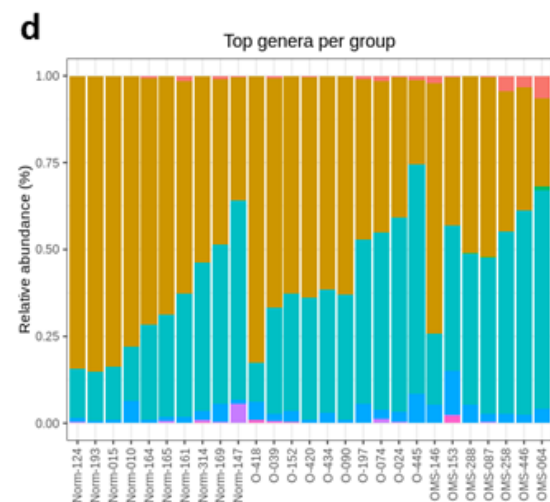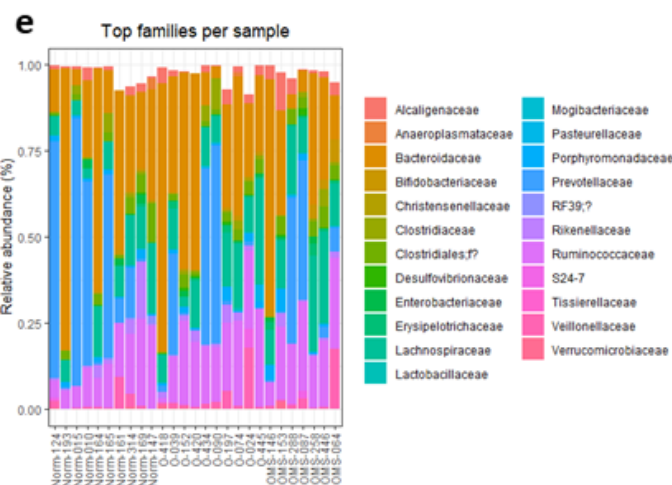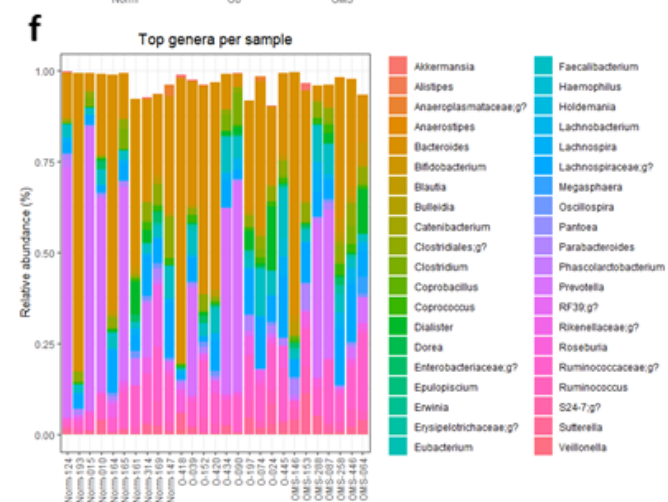

Supplement: Supplementary file 3 — Additional file 3: Figure S2. Top ten most abundant taxa: Per group a) phyla, b) families and c) genera per group; and per sample d) phyla, e) families and f) genera. NW = Normal Weight, O = Obese, and OMS = Obese with Metabolic Syndrome. [file 12934_2020_1319_MOESM3_ESM.pdf]

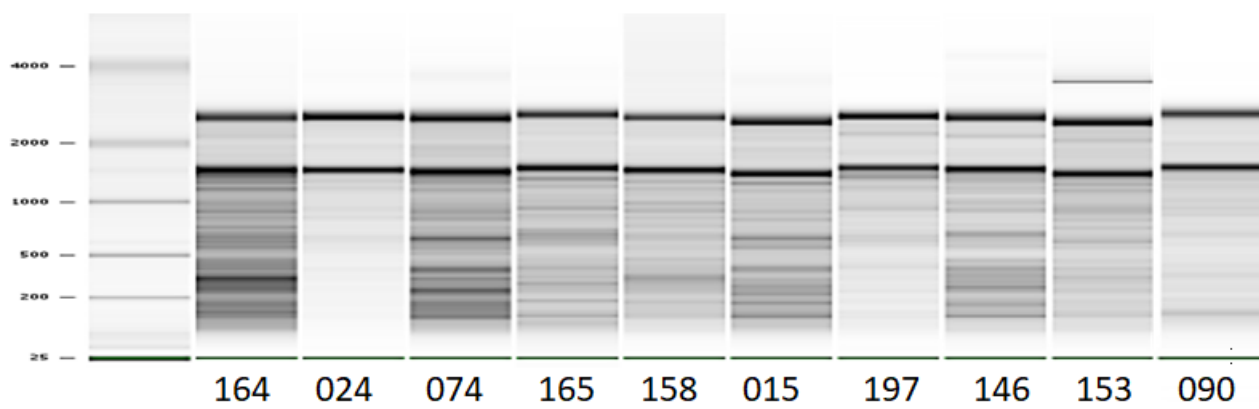

Supplement: Supplementary file 6 — Additional file 6: Figure S4. Bioanalyzer profile of each sample used for the metatranscriptome. [file 12934_2020_1319_MOESM6_ESM.pdf]

## a Molecular Function

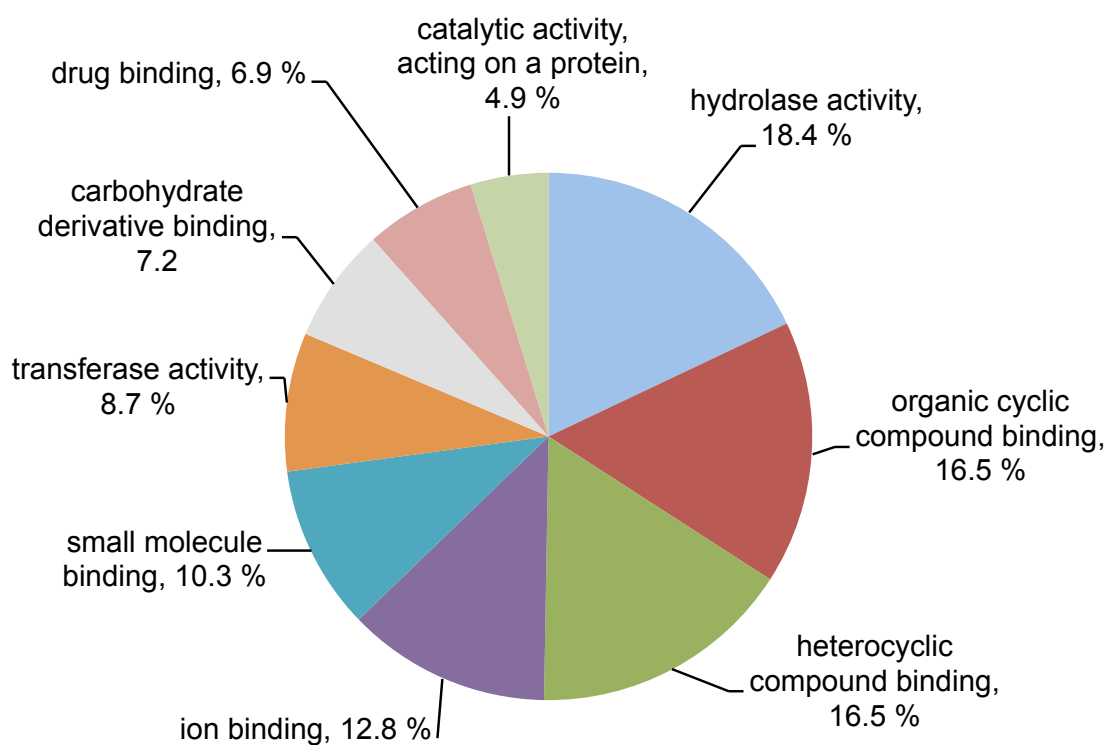

## b Cellular Component

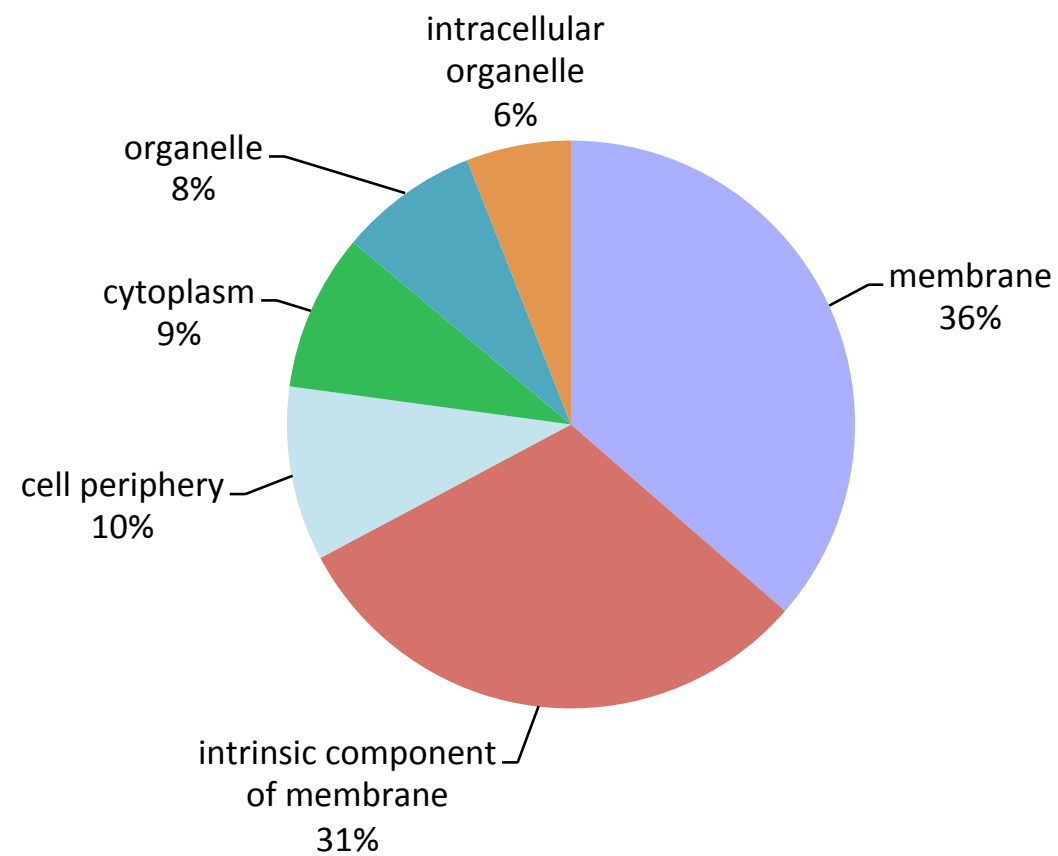

## c Biological Process

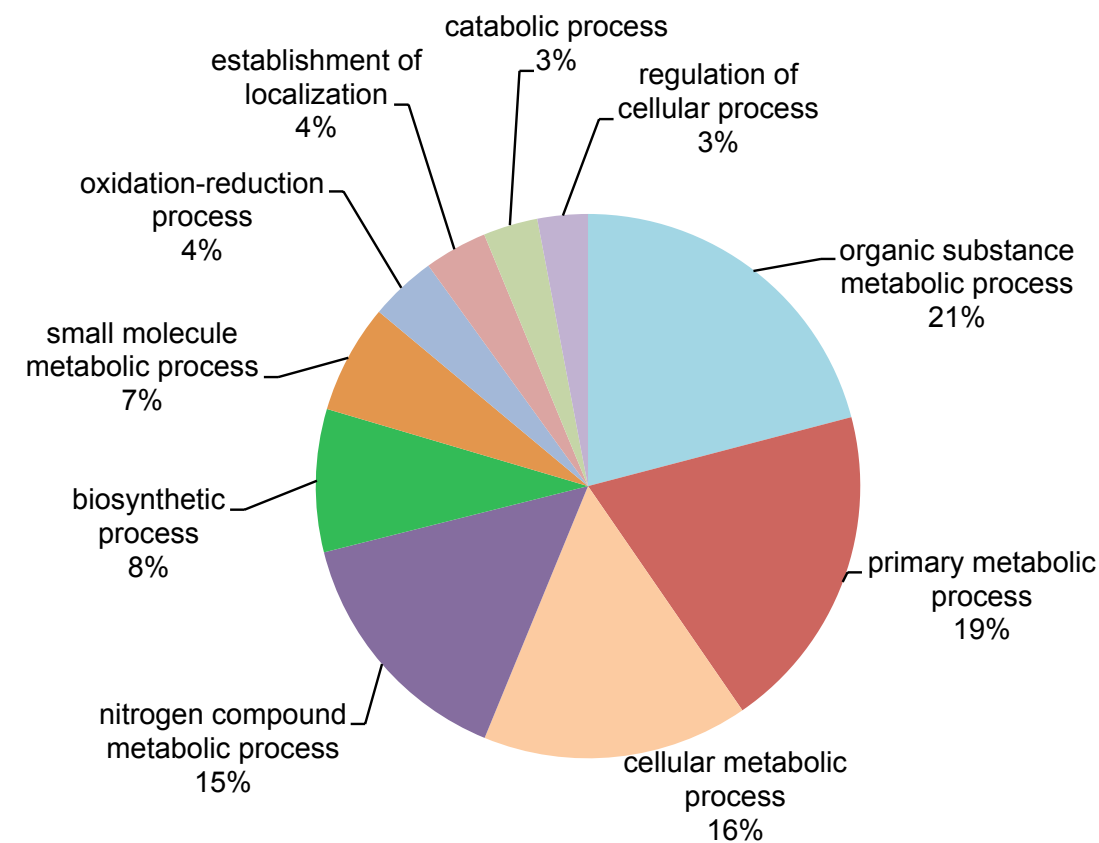

## d Enzymes

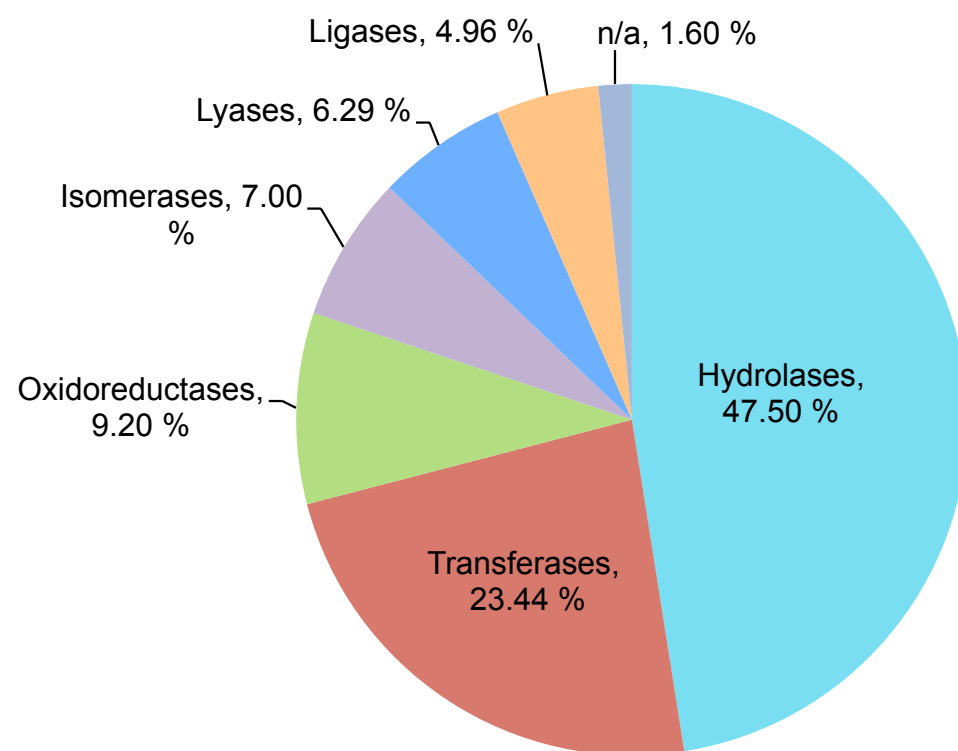

Supplement: Supplementary file 8 — Additional file 8: Figure S5. GO terms and Enzyme commission class distribution of the total ES proteins. Each pay graph shows the different GO and enzyme terms associated with the complete ES proteins encoded in the metatranscriptome for a) Molecular Function, b) Cellular Component, c) Biological Process, and d) Enzyme categories. [file 12934_2020_1319_MOESM8_ESM.pdf]

**a**

## Recruitment of Unique GO per Sample

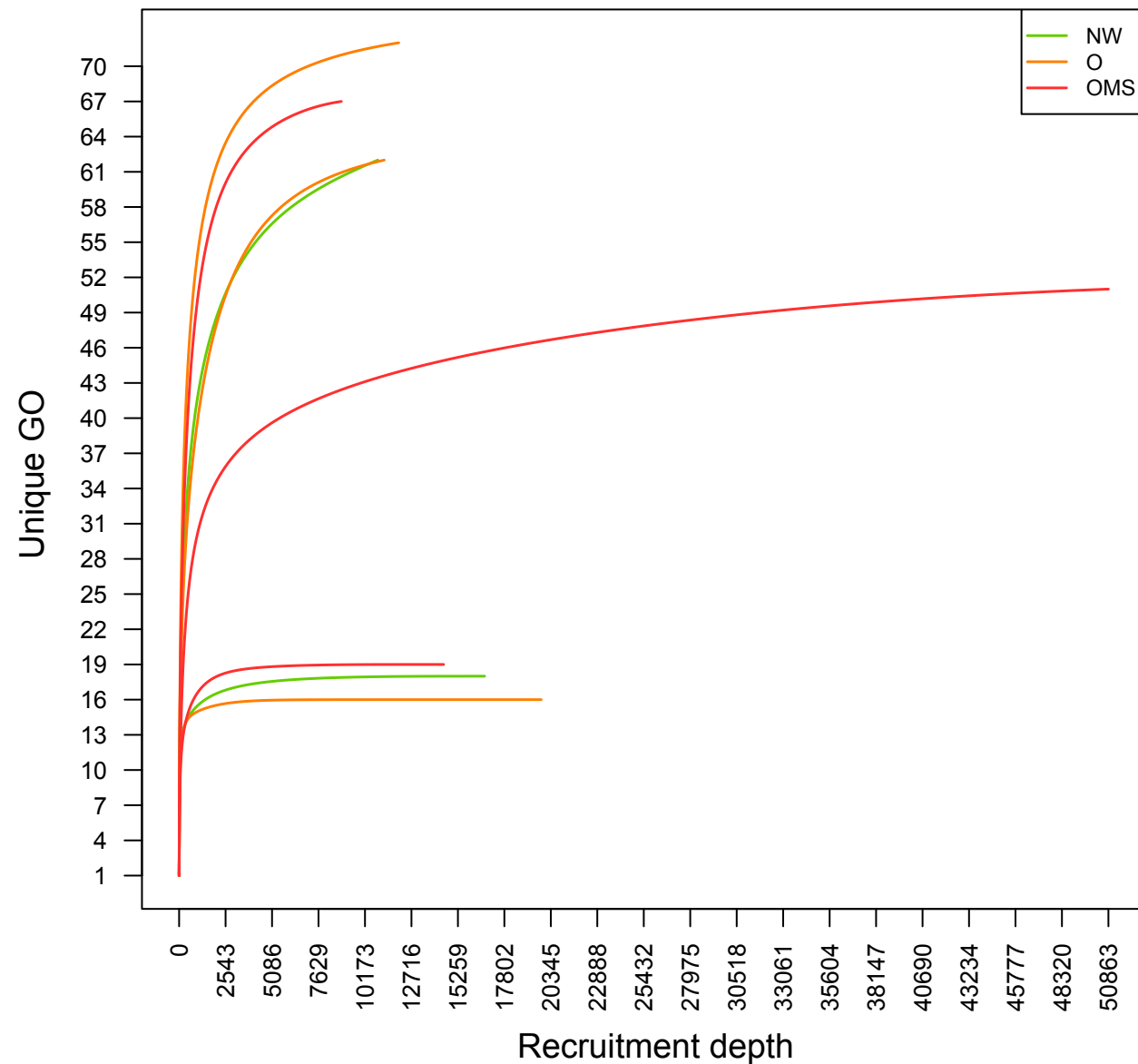**b**

## Recruitment of Unique EC per Sample

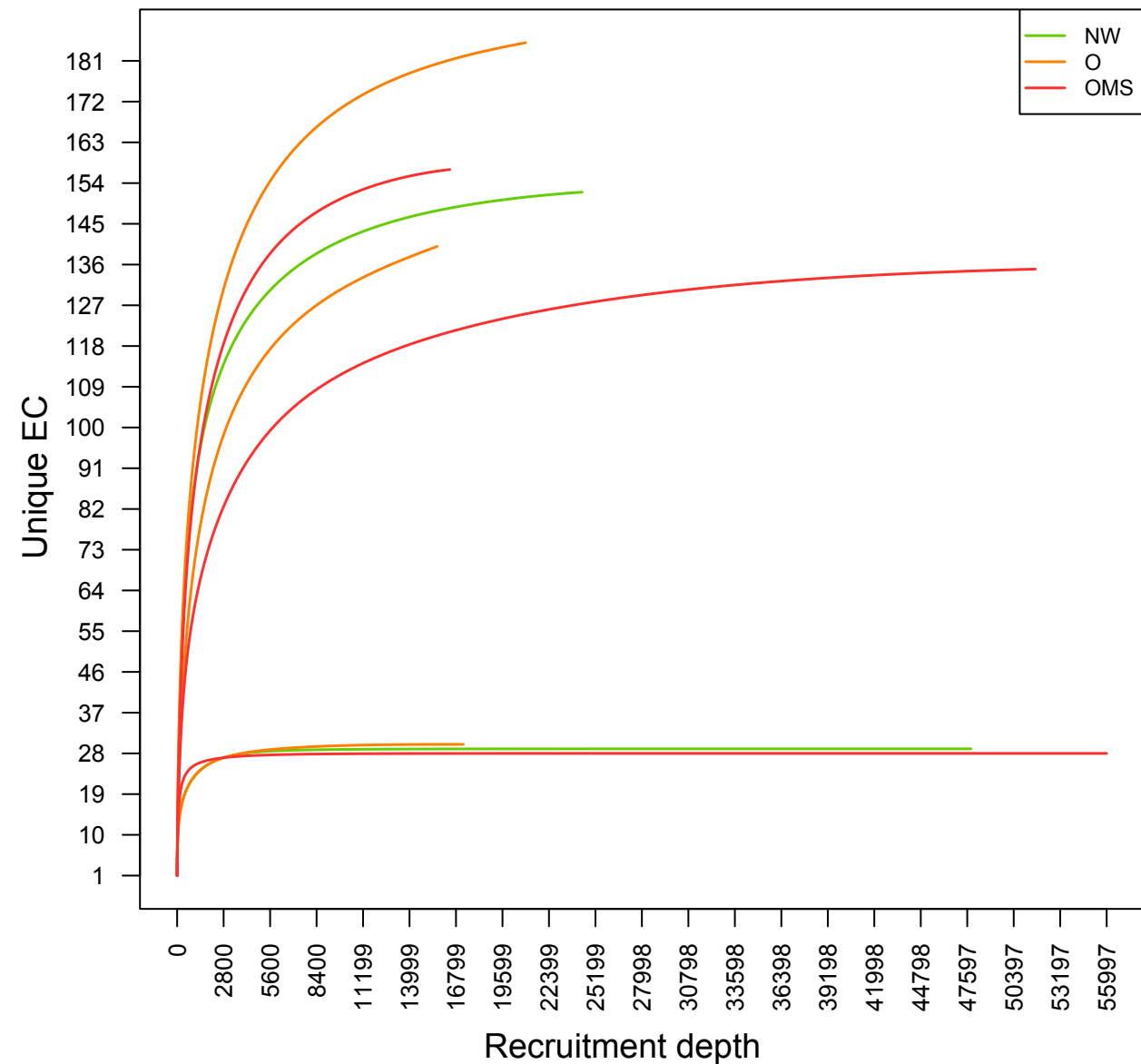

Supplement: Supplementary file 9 — Additional file 9: Figure S6. Recruitment of unique functional categories per sample. Samples are colored by NW, O, and OMS groups. a) Recruitment of unique Gene Ontology (GO) features in progressive randomized no-replacement rarefactions based on Hurlbert calculations. b) Recruitment of unique Enzyme Commission numbers (EC) in progressive randomized no-replacement rarefactions based on Hurlbert calculations. [file 12934_2020_1319_MOESM9_ESM.pdf]

Expression signal in per sample

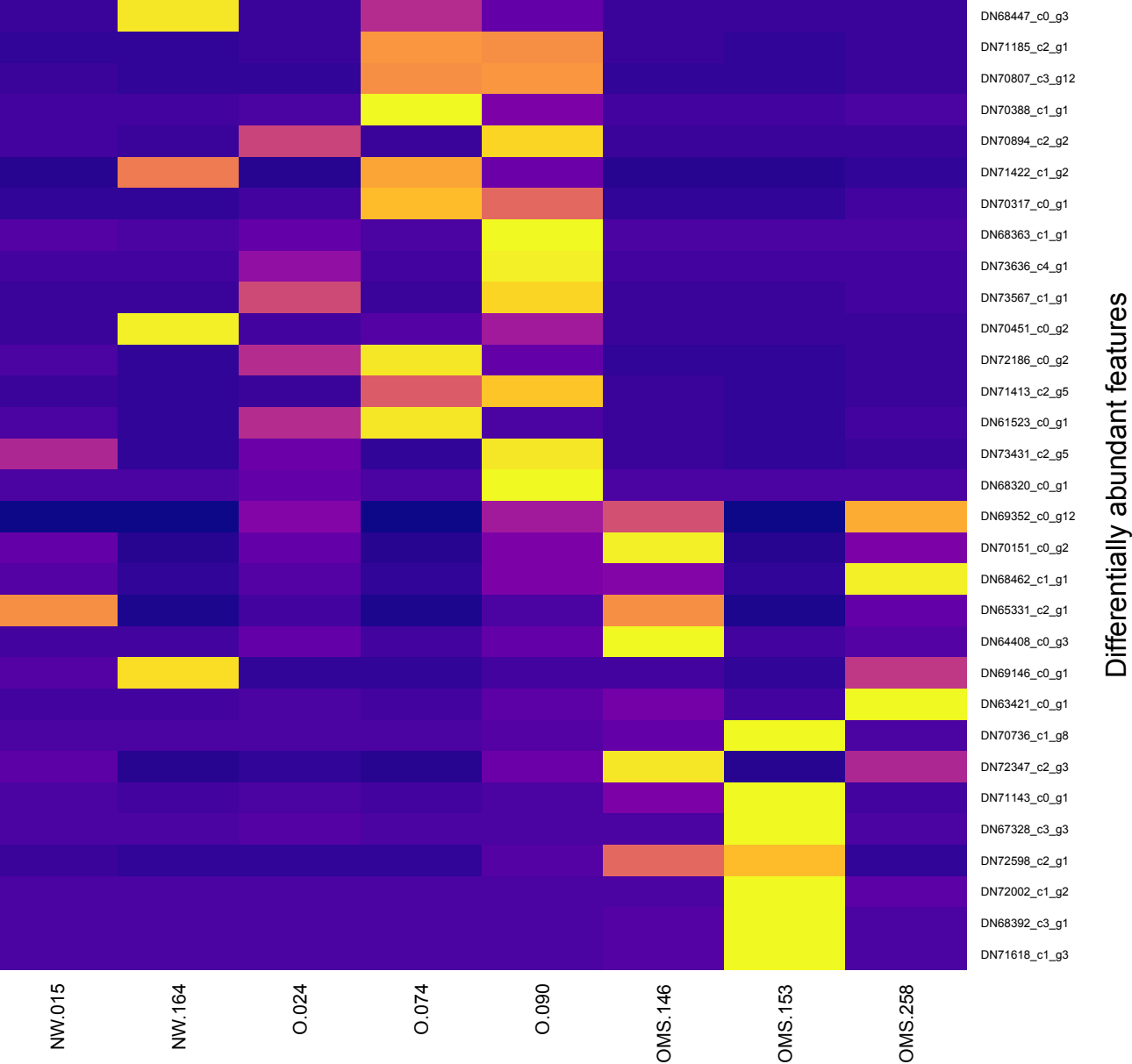

Supplement: Supplementary file 10 — Additional file 10: Figure S7. Per-sample expression of transcripts associated with the O and OMS groups. The total normalized RSEM abundance of each transcript per sample with a higher abundance in yellow. Only differentially abundant transcripts associated with the case groups are shown. DESeq-based standardization of the expression signal was carried out considering all samples. [file 12934_2020_1319_MOESM10_ESM.pdf]

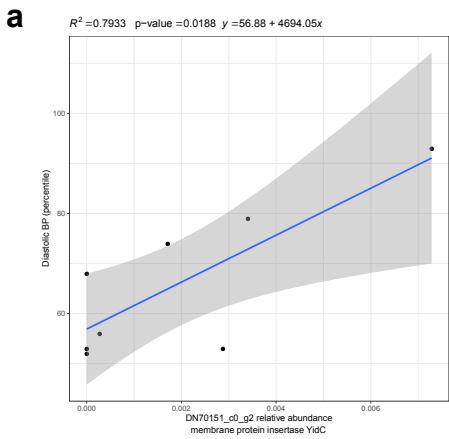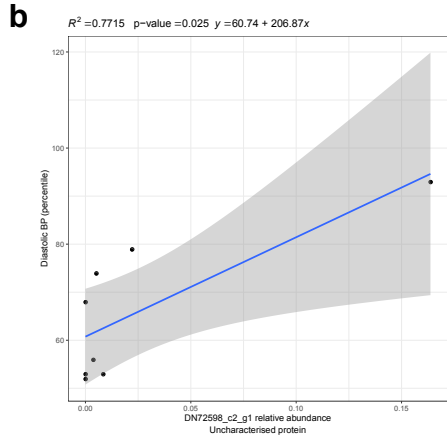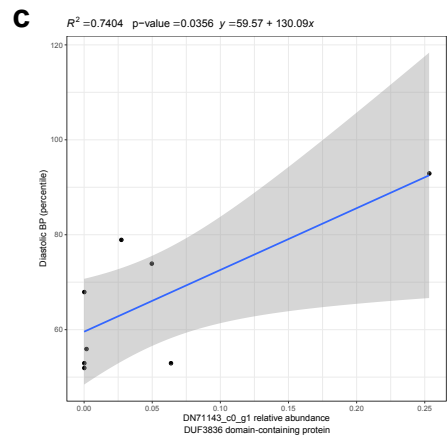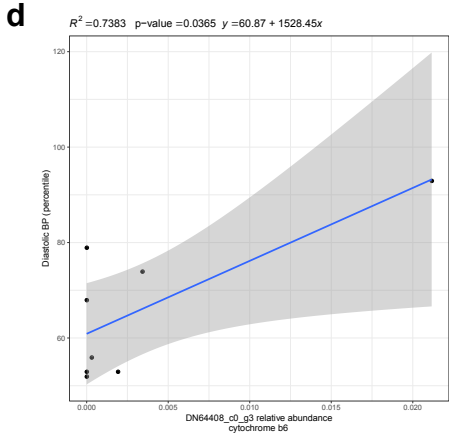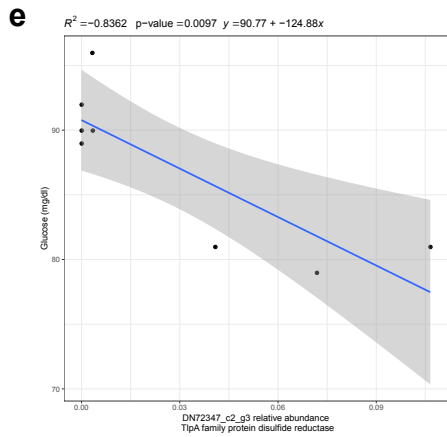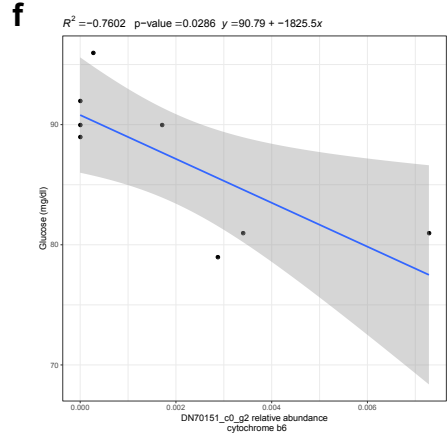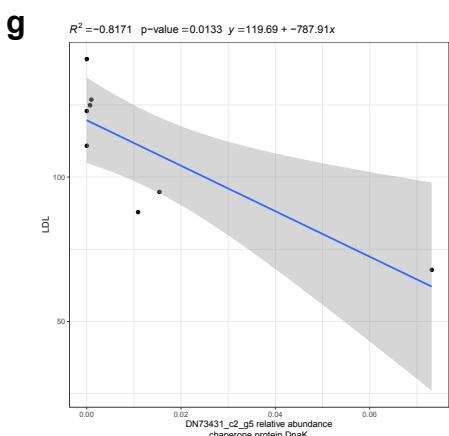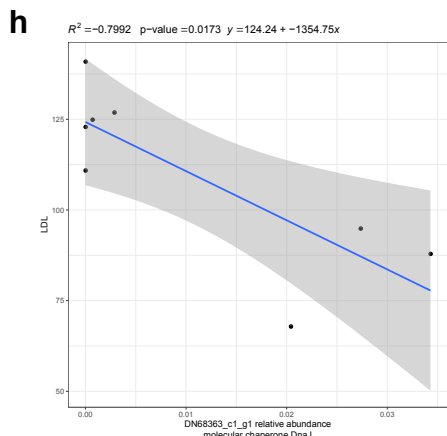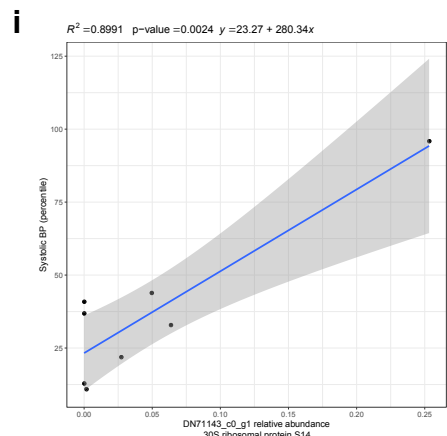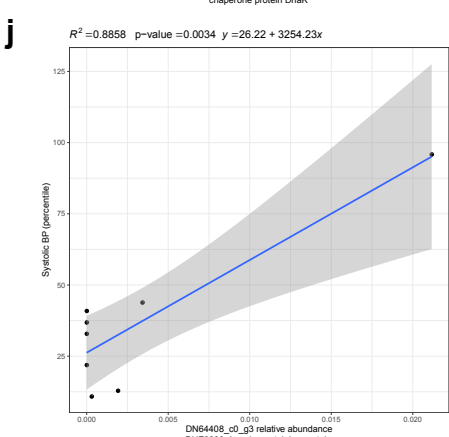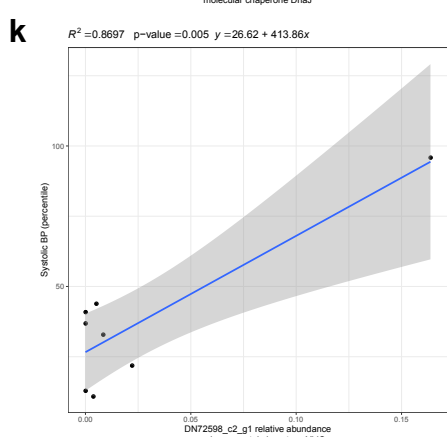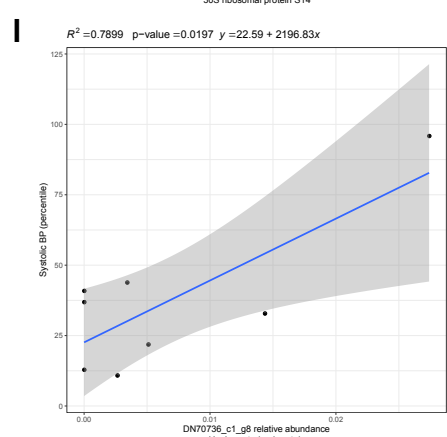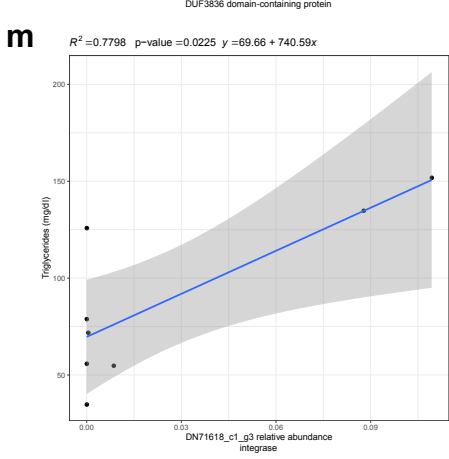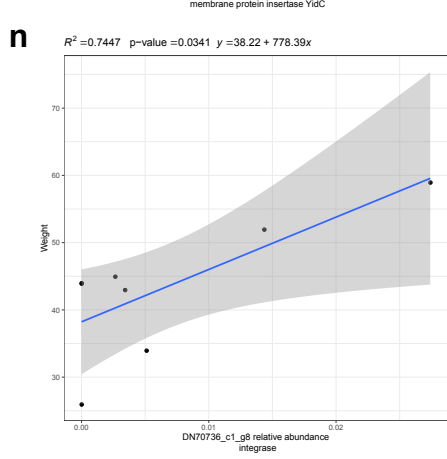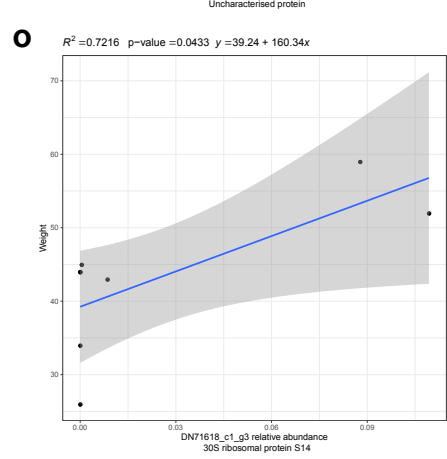

Supplement: Supplementary file 11 — Additional file 11: Figure S8. Correlations between differentially abundant transcripts and clinical data. Only significant correlations are shown (α = 0.05). Pearson correlation, the p-value is shown. A linear regression model is fitted to the data using the transcript as a predictor; the intercept and slope are shown, and SE is presented as a shadow. Transcripts (x-axis) are shown as relative normalized RSEM abundances. The scale of the clinical data is different for each parameter. a–d) Transcripts positively correlating with diastolic blood pressure (percentile). e–f) Transcripts negatively correlating with glucose levels (mg/dL). g–h) Transcripts negatively correlating with LDL levels (mg/dL). i–l) Transcripts positively correlating with systolic blood pressure (percentile). m) Transcripts positively correlated to triglyceride levels (mg/dL). n–o) Transcripts positively correlated to subject weight (g). [file 12934_2020_1319_MOESM11_ESM.pdf]

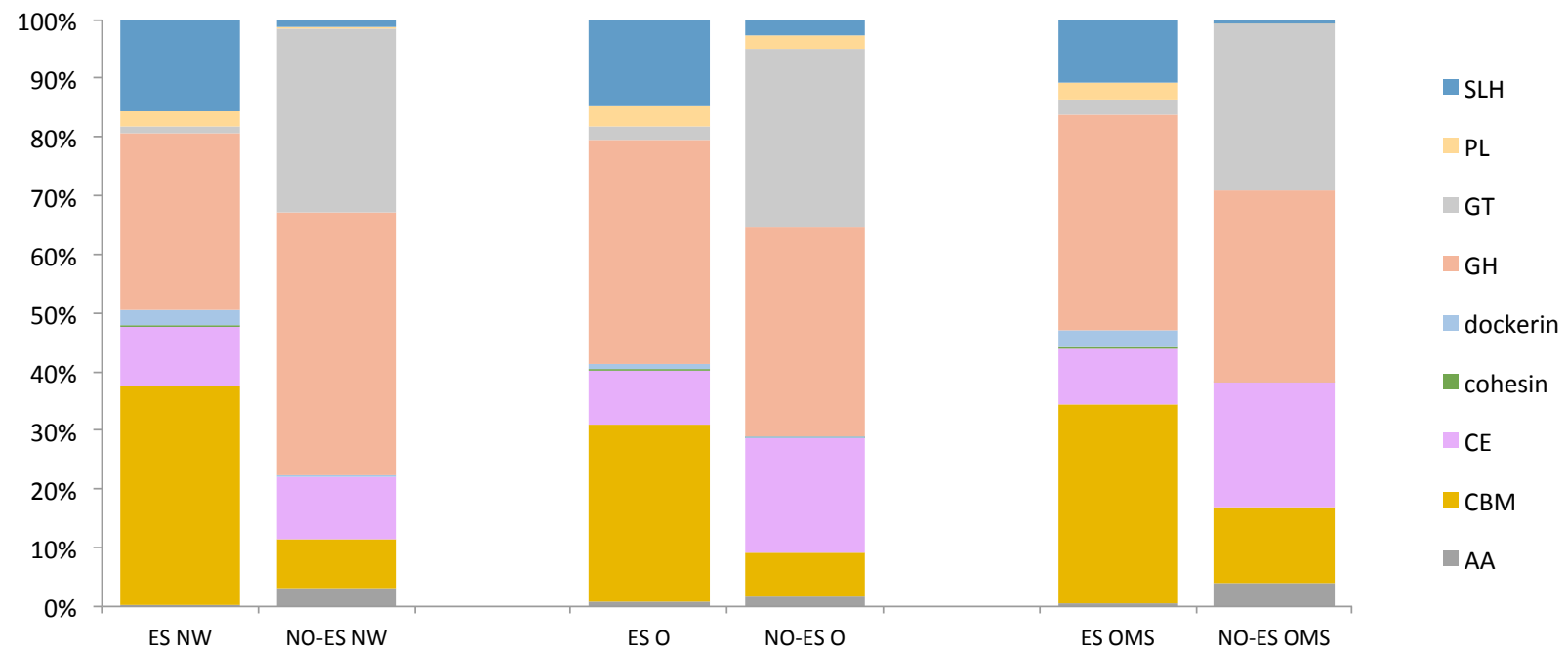

Supplement: Supplementary file 12 — Additional file 12: Figure S9. Carbohydrate-active enzyme distribution. Relative abundance of CAZy enzyme families across the secreted and non-secreted proteins in the NW, O, and OMS groups. [file 12934_2020_1319_MOESM12_ESM.pdf]
