# Supplementary material for: Metatranscriptomic analysis to define the Secrebiome, and 16S rRNA profiling of the gut microbiome in obesity and metabolic syndrome of Mexican children
Source: Microb Cell Fact. 2020 Mar 6;19:61. doi: 10.1186/s12934-020-01319-y (PMC7060530; doi:10.1186/s12934-020-01319-y)

PCoA - Weighted UniFrac

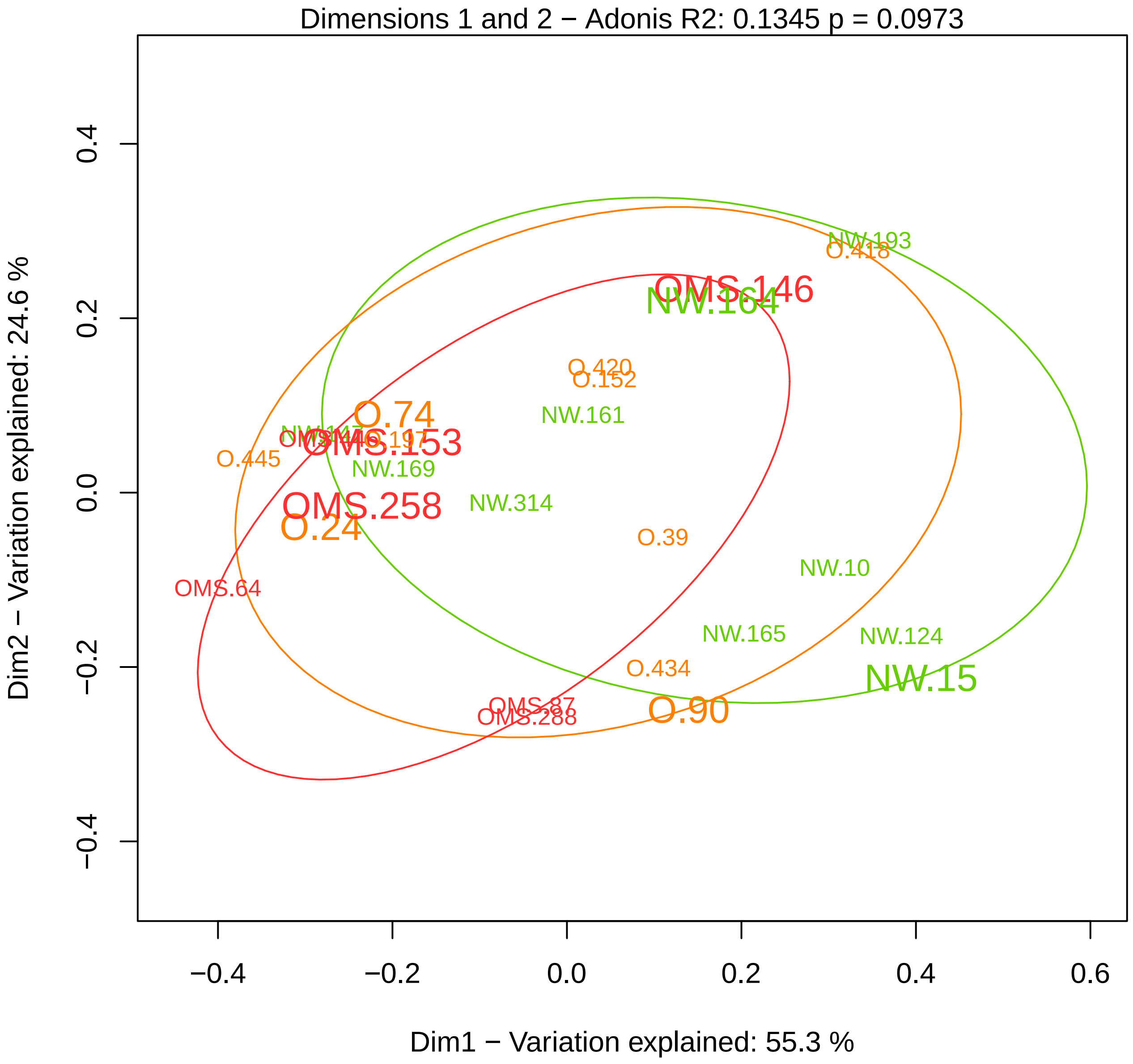

Supplement: Supplementary file 4 — Additional file 4: Figure S3. Principal coordinate analyses of Weighted UniFrac distances. Distances are based on taxa abundance. The two linear combinations explaining the most variation are shown as dimensions 1 and 2. Elipses were calculated based on the most distant samples per group. Samples for which RNA-seq information is available are presented with a larger font. [file 12934_2020_1319_MOESM4_ESM.pdf]
