# Supplementary material for: Metatranscriptomic analysis to define the Secrebiome, and 16S rRNA profiling of the gut microbiome in obesity and metabolic syndrome of Mexican children
Source: Microb Cell Fact. 2020 Mar 6;19:61. doi: 10.1186/s12934-020-01319-y (PMC7060530; doi:10.1186/s12934-020-01319-y)
Supplement: Supplementary file 7 — Additional file 7: Table S3. Basic statistics of the metatranscriptome assembly. [file 12934_2020_1319_MOESM7_ESM.pdf]

**Table S3.** Basic statistics of the metatranscriptome assembly

| | |
|---|---|
| Counts of transcripts:<br><br>Total trinity 'genes': 224,427<br>Total trinity transcripts: 309,550<br>Percent GC: 50.32 | |
| Stats based on ALL transcript contigs:<br><br>Contig N10: 3,980<br>Contig N20: 2,248<br>Contig N30: 1,527<br>Contig N40: 1,108<br>Contig N50: 811 | Median contig length: 359<br>Average contig: 605.01<br>Total assembled bases: 187,279,659 |
| Stats based on ONLY LONGEST ISOFORM per 'GENE':<br><br>Contig N10: 3,908<br>Contig N20: 2,186<br>Contig N30: 1,430<br>Contig N40: 990<br>Contig N50: 702 | Median contig length: 333<br>Average contig: 557.04<br>Total assembled bases: 125,015,187 |
